# RNA binding protein ZFP36L1 promotes ferroptosis in chronic rhinosinusitis by destabilizing CAMK2A mRNA and impairing mitochondrial quality control

**DOI:** 10.1002/ctm2.70661

**Published:** 2026-04-29

**Authors:** Jiayi Xiong, Shihan Zhang, Chunhua Li, Yufeng Ai, Xinru Liu, Yuxiang Liu, Hongbing Liu

**Affiliations:** ^1^ Department of Otolaryngology Head and Neck Surgery The Second Affiliated Hospital Jiangxi Medical College Nanchang University Nanchang China

**Keywords:** CAMK2A, chronic rhinosinusitis, ferroptosis, mitochondrial quality control, RNA‐binding proteins, ZFP36L1

## Abstract

**Background:**

RNA‐binding proteins (RBPs) and ferroptosis have been demonstrated to play important roles in the progression of chronic rhinosinusitis (CRS). However, the regulatory mechanisms underlying the interaction between RBPs and ferroptosis in CRS, particularly regarding mitochondrial metabolism, remain elusive.

**Methods:**

Hub genes correlated with RBP‐related genes, ferroptosis‐related genes and mitochondrial‐related genes were identified by integrated bioinformatics analysis. CRS in vivo models were constructed, clinical samples were collected, and mechanistic analyses were performed for validation.

**Results:**

*ZFP36L1* was identified as the hub gene associated with CRS development. In vivo experiments demonstrated that *ZFP36L1* directly binds to the 3′‐untranslated region of *CAMK2A* mRNA and promotes its degradation through AU‐rich element recognition. *ZFP36L1* knockout in CRS mouse models restored *CAMK2A* expression and significantly attenuated ferroptosis markers, reactive oxygen species accumulation and mitochondrial dysfunction. Rescue experiments revealed that *CAMK2A* knockdown reversed the protective effects of *ZFP36L1* depletion on ferroptosis and mitochondrial quality control. Clinical samples confirmed that ZFP36L1 expression was inversely correlated with CAMK2A levels, and both were associated with disease severity.

**Conclusion:**

This study identifies *ZFP36L1–CAMK2A* as a contributory regulatory mechanism in CRS pathogenesis. *ZFP36L1* promotes ferroptosis by destabilizing *CAMK2A* mRNA, leading to mitochondrial dysfunction and subsequent epithelial cell death. These findings provide new mechanistic insights into CRS progression and identify potential therapeutic targets.

**Highlights:**

- ZFP36L1 is identified as a key driver gene in chronic rhinosinusitis (CRS) progression via integrated bioinformatics analysis.
- ZFP36L1 promotes ferroptosis by binding to and destabilizing CAMK2A mRNA through AU‐rich elements in its 3'‐UTR.
- Genetic knockout of ZFP36L1 attenuates ferroptosis and restores mitochondrial quality control in CRS models.
- Clinical validation confirms the ZFP36L1‐CAMK2A axis correlates with disease severity and represents a potential therapeutic target.

## Graphical abstract

This graphical abstract illustrates the novel *ZFP36L1–CAMK2A* regulatory axis in chronic rhinosinusitis (CRS) pathogenesis:

1. **Molecular initiation**: In nasal epithelial cells, the RNA‐binding protein ZFP36L1 (upregulated in CRS) directly binds to AU‐rich elements (AREs) in the 3′‐untranslated region (3′‐UTR) of *CAMK2A* mRNA, promoting its degradation and leading to reduced CAMK2A protein expression.

2. **Cellular dysfunction**: The downregulation of *CAMK2A* impairs mitochondrial quality control, resulting in mitochondrial dysfunction (evidenced by swollen morphology, decreased membrane potential, reduced adenosine triphosphate (ATP) production and elevated ROS). This mitochondrial damage drives the accumulation of lipid ROS and iron (Fe^2+^), ultimately inducing ferroptosis.

3. **Clinical correlation**: In human clinical samples, a progressive increase in *ZFP36L1* expression and a reciprocal decrease in *CAMK2A* expression are observed from healthy controls (HCs) to CRS without nasal polyps (CRSsNP) and to CRS with nasal polyps (CRSwNP), correlating with disease severity.

Conclusion: The ZFP36L1–CAMK2A‐ferroptosis axis is a key mechanistic pathway contributing to CRS progression, highlighting potential therapeutic targets.

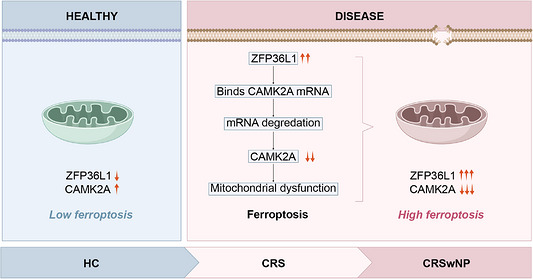

## 1 INTRODUCTION

Chronic rhinosinusitis (CRS) is a persistent inflammatory disease of the nasal cavity and paranasal sinuses characterized by nasal obstruction and purulent secretion that has troubled patients for a long time.^1^ Clinically, CRS is subdivided into CRS with nasal polyps (CRSwNP) and CRS without nasal polyps (CRSsNP), a distinction based primarily on the existence or absence of nasal polyps.^2^ Despite advances in medical and surgical treatments, CRS management remains challenging. Current therapies, including topical corticosteroids and endoscopic sinus surgery, have significant limitations: Long‐term corticosteroid use is associated with adverse effects and potential drug resistance. These therapeutic challenges underscore the urgent need to identify novel molecular targets and develop more effective treatment strategies for CRS.

RNA‐binding proteins (RBPs) are key regulators involved in RNA processing, transport and stability, which in turn affect cell differentiation and function.^3^ In CRS, dysregulation of RBP‐related genes (RBPRGs) is closely associated with abnormal functional processes and cell differentiation in nasal mucosal cells resulting from persistent inflammatory stimulation.^4^, ^5^ Moreover, aberrant expression of RBPRGs is linked to abnormal cell proliferation, apoptosis and differentiation and contributes to nasal polyp formation in CRS patients.^5^

Ferroptosis is an iron‐dependent form of programmed cell death distinct from apoptosis, characterized by molecular and morphological changes that have been documented in nasal tissues of CRSwNP patients.^6^ Previous studies have identified multiple ferroptosis‐related genes (FRGs) in CRS pathology; for instance, *ALOX1*5 and acyl‐CoA synthetase long‐chain family member 4 (ACSL4) were highly expressed, whereas glutathione peroxidase 4 (*GPX4*) was downregulated in CRSwNP patients.^7^ Furthermore, FRGs also regulate immune cells’ inflammatory responses, thereby influencing CRS progression.^8^ Notably, RBPs can bind to specific mRNAs, thereby affecting the stability and translation efficiency of FRGs, and participate in regulating multiple signalling pathways closely related to ferroptosis mechanisms.^9^, ^10^, ^11^ For example, RBMS1 was identified as a translation promoter of solute carrier family 7 member 11 (*SLC7A11*) in lung cancer, and its deletion enhances ferroptosis, thereby inhibiting lung cancer cell growth.^10^ These findings suggest a direct and important role for RBPs in ferroptosis regulation. However, the regulatory mechanisms governing the interaction between RBPs and ferroptosis in CRS, particularly regarding mitochondrial metabolism, remain elusive.

Additionally, RBPs participate in mitochondrial genome replication and mediate the translational balance between cytoplasm and mitochondria to modulate mitochondrial respiratory capacity.^12^ Mitochondrial dysfunction has been demonstrated to be significantly associated with the occurrence and development of multiple inflammation‐related diseases.^13^, ^14^ Specifically, altered mitochondrial functions in epithelial cells have been implicated in CRSwNP pathogenesis,^15^ with the Akt/mTOR pathway playing a crucial role in this process.^16^ However, the regulatory mechanisms by which RBPs modulate mitochondrial function in CRS remain unclear.

Therefore, this study aimed to investigate the regulatory role of the RBP ZFP36L1 in CRS inflammatory progression. We sought to identify the direct downstream targets of ZFP36L1 and elucidate the molecular mechanisms by which ZFP36L1 regulates ferroptosis and mitochondrial quality control in nasal mucosal epithelial cells. Our findings may provide novel mechanistic insights into CRS pathogenesis and identify potential therapeutic targets.

## 2 MATERIALS AND METHODS

### 2.1 Data extraction and pretreatment

CRS‐related datasets were downloaded from the Gene Expression Omnibus (GEO) database. The GSE136825 dataset contained 42 CRS and 33 healthy control (HC) samples from nasal polyp tissues, whereas the GSE179265 dataset contained 17 CRS and 7 HC samples from nasal tissues. The two datasets were combined, and batch effects were removed using the “sva” R package,^17^ resulting in a combined dataset of 59 CRS and 40 HC samples. In parallel, a total of 564 FRGs were obtained from the FerrDbV2 database (zhounan.org/ferrdb/curr), 1136 human mitochondrial‐related genes (MRGs) were retrieved from the MitoCarta3.0 database (https://www.broadinstitute.org/mitocarta), and 416 RBPRGs were downloaded from the Ensembl website (https://www.ensembl.org/).

### 2.2 Screening for hub genes associated with CRS

The “limma” R package was utilized to identify differentially expressed genes (DEGs) between the 59 CRS and 40 HC samples in the integrated dataset (*p* < .05).^18^ Subsequently, a weighted gene co‐expression network was constructed via the “WGCNA” R package. In this step, the scores for FRGs, MRGs and RBPRGs served as traits to screen for modules significantly associated with these traits.^19^ Target genes were derived by taking the intersection of the DEGs and the relevant module genes using the “venn” function. Based on the target genes identified above, characteristic genes were calculated using three machine learning algorithms: least absolute shrinkage and selection operator (LASSO), support vector machine recursive feature elimination (SVM‐RFE) and random forest (RF). Hub genes were defined as the overlapping characteristic genes identified by all three algorithms. Receiver operating characteristic (ROC) curves of the hub genes were generated using the “pROC” R package to assess the diagnostic ability to distinguish CRS from HC. Subsequently, Mendelian randomization (MR) analysis was performed to identify hub genes with causal relationships with CRS. Hub genes meeting both criteria—area under the ROC curve (AUC) > .7 and evidence of causal relationship with CRS by MR analysis—were defined as key genes for CRS.

### 2.3 Construction of the in vivo CRS animal model

Male C57BL/6 mice (6–8 weeks old) were randomly divided into two groups: the wild‐type (WT)‐NC (WT control, *n* = 10) and the WT‐lipopolysaccharide (LPS) (CRS model, *n* = 10). The CRS model was established by nasal instillation of LPS (5 µg/g per dose) on Days 1, 8 and 15. The WT‐NC group received nasal instillation of physiological saline (.9% NaCl) on the same schedule. All mice were fasted for 1 day before cervical dislocation. Transcript and protein expression levels of *ZFP36L1* were detected using quantitative reverse transcription polymerase chain reaction (qRT‐PCR) and western blotting (WB) to assess the expression patterns of ZFP36L1 in mice. Subsequently, *ZFP36L1* knockout mouse models were established to analyse the effects of *ZFP36L1* on mitochondrial mass and ferroptosis in CRS. Specifically, sgRNA sequences targeting the *ZFP36L1* exon 2 (5′‐GATCTTCTGGGAGCTGGAGTGG‐3′) were designed and validated. The validated sgRNA/Cas9 vector was microinjected into mouse zygotes. F0 generation mice with positive gene knockdown were screened by PCR, and F1 generation mice were bred for experiments. *ZFP36L1* knockout WT and WT‐LPS mouse models were then established using the method described above. Histopathological changes of the nasal mucosa were assessed by haematoxylin and eosin (HE) staining and periodic acid–Schiff (PAS) staining. Histopathological severity was evaluated using a blinded semi‐quantitative scoring system. Epithelial hyperplasia was independently scored on a 0–3 scale. Serum levels of inflammatory factors (TNF‐α, TNF‐γ and IL‐32) were measured by enzyme‐linked immunosorbent assay.

### 2.4 Regulatory relationship analysis between *ZFP36L1* and ferroptosis in CRS

The experiment comprised four groups: WT‐NC (*n* = 10), WT‐LPS (*n* = 10), ZFP36L1^KO^‐WT (*n* = 10) and ZFP36L1^KO^‐LPS (*n* = 10) groups. Ferroptosis‐related proteins, including transferrin receptor (TFR), *GPX4, ACSL4*, ferritin heavy chain (*FTH1*) and *SLC7A11*, in nasal mucosal tissue were detected by qRT‐PCR and WB. Second, 2′,7′‐dichlorodihydrofluorescein diacetate (*DCFH‐DA*) combined with flow cytometry was used for detecting the content of reactive oxygen species (ROS) in nasal mucosal epithelial cells of mice. Third, the content of iron (II) ion (Fe^2+^) in the nasal mucosa of each group of mice was detected by FerroOrange (Dojindo) kit. In addition, the redox reaction is a key link in ferroptosis; therefore, the contents of glutathione (GSH), oxidized glutathione (GSSG) and malondialdehyde (MDA) in each group were further examined to study the regulation of ferroptosis by ZFP36L1 in nasal mucosal epithelial cells.

### 2.5 Regulatory relationship analysis between ZFP36L1 and mitochondrial quality control in CRS

The experiment was divided into four groups: WT‐NC (*n* = 10), WT‐LPS (*n* = 10), *ZFP36L1* ^KO^‐WT (*n* = 10) and *ZFP36L1* ^KO^‐LPS (*n* = 10). First, the number and morphology changes of mitochondria in each group were assessed by Mito‐Tracker and Hoechst 33342 colocalization staining under the laser confocal. Mitochondrial mean areas (µm^2^) were quantified using ImageJ. Then, the ultrastructural changes of mitochondria in nasal mucosal tissues of experimental mice were observed by transmission electron microscopy, and JC‐1 probe was applied to observe the changes in the mitochondrial transmembrane potential. For TEM quantification, mitochondrial morphology was scored on a 0–3 scale for membrane intactness. Red/green fluorescence ratio was calculated by flow cytometry (BD FACSCanto II) using PE and FITC channels. Immediately thereafter, the activity of cytochrome *c* oxidase (DCytaa3) and the situation of the mitochondrial adenosine triphosphate (ATP) generation were measured using the mitochondrial DCytaa3 activity assay kit and the ATP generation assay kit. In addition, the transcript and protein expression levels of the biomarkers of mitochondrial biogenesis (PGC‐1α and Nrf1), mitochondrial quality control (OPA1 and Drp1) and mitophagy (LC3I and Bcl‐2) were detected by qRT‐PCR and WB. Finally, the situation of energy metabolism in hepatocytes was detected by Seahorse energy metabolism analyser.

### 2.6 Analysis of the effect of the regulatory relationship between the key gene and ferroptosis on mitochondrial quality control

Ferroptosis derivant (erastin) and inhibitor (ferrostatin‐1, Fer‐1) were used to modulate the four mouse models (WT‐NC, WT‐LPS, *ZFP36L1* ^KO^‐WT and *ZFP36L1* ^KO^‐LPS) to explore the role of ferroptosis, regulated by the key gene, in mitochondrial biogenesis. Specifically, after successful establishment of the four models, a 20 µL injection containing either 10 mg/mL erastin or 1 mg/mL Fer‐1 in soliquoid (5% dimethyl sulfoxide, 5% Tween‐80, 40% polyethylene glycol‐400 and 50% .9% NaCl) was administered intravenously every 12 h for four doses.^20^ All mice were fasted for 1 day before cervical dislocation. The experiment comprised 12 groups: WT‐NC (*n* = 10), WT‐LPS (*n* = 10), *ZFP36L1*KO‐WT (*n* = 10), *ZFP36L1*KO‐LPS (*n* = 10), WT+erastin (*n* = 10), LPS+erastin (*n* = 10), *ZFP36L1*KO‐WT+erastin (*n* = 10), *ZFP36L1*KO‐LPS+erastin (*n* = 10), WT+Fer‐1 (*n* = 10), LPS+Fer‐1 (*n* = 10), *ZFP36L1*KO‐WT+Fer‐1 (*n* = 10) and *ZFP36L1*KO‐LPS+Fer‐1 (*n* = 10). For all groups, mitochondrial morphology, ultrastructure, cytochrome *c* oxidase activity, ATP generation, and biomarkers of mitochondrial biogenesis, mitochondrial quality control and mitophagy were assessed. Additionally, energy metabolism status was evaluated to examine the relationship between the ZFP36L1‐ferroptosis interaction and the mitochondrial quality control system.

### 2.7 Mechanistic analysis of ZFP36L1 regulation of CAMK2A in CRS

A *ZFP36L1* overexpression‐related dataset (GSE79633) was downloaded from the GEO database. The intersection of DEGs and module genes yielded the target genes. Next, a protein–protein interaction (PPI) network was established via the “STRING” database (score > .9), and network nodes were analysed using the MCC algorithm.^21^ The expression of core genes in the PPI network was detected in the combined dataset, and correlations between ZFP36L1 and core genes were analysed using Spearman's correlation test. Furthermore, expression of ZFP36L1 and its core downstream genes in the animal models (WT, WT‐LPS, *ZFP36L1* ^KO^‐WT and ZFP36L1^KO^‐LPS) was detected, and colocalization relationships were visualized by multiplex immunofluorescence.

### 2.8 RNA immunoprecipitation (RIP) assay

The direct interaction between the ZFP36L1 protein and CAMK2A mRNA was validated through RNA immunoprecipitation (RIP) assays, which were carried out with the Magna RIP RBP Immunoprecipitation Kit (Millipore) in adherence to the manufacturer's protocol. In short, nasal epithelial cells underwent lysis using RIP lysis buffer. The resulting lysates were then incubated overnight at 4°C with magnetic beads bound to an anti‐ZFP36L1 antibody (Sigma‐Aldrich, T5327) or a control IgG. Following a washing step, RNA was isolated from the immunoprecipitates using TRIzol reagent and subjected to RT‐PCR or qRT‐PCR analysis. Glyceraldehyde‐3‐Phosphate Dehydrogenase (GAPDH) mRNA was used as a negative control. Finally, we quantified the enrichment of CAMK2A mRNA in the ZFP36L1 immunoprecipitates relative to the IgG controls.

### 2.9 mRNA stability assay

To determine whether *ZFP36L1* affects *CAMK2A* mRNA stability, nasal epithelial cells were transfected with *ZFP36L1* knockdown (sh*ZFP36L1*), overexpression (oe*ZFP36L1*) or control vectors for 48 h. Cells were then treated with actinomycin D (5 µg/mL, Sigma‐Aldrich) to block new RNA synthesis. Total RNA was extracted at 0, 1, 2, 3 and 4 h after actinomycin D treatment, and CAMK2A mRNA levels were measured by qRT‐PCR. The relative mRNA levels were normalized to the 0‐h time point, and mRNA decay curves were plotted. The half‐life (*t*) of CAMK2A mRNA was calculated for each experimental group.

### 2.10 Dual‐luciferase reporter assay

To investigate whether *ZFP36L1* regulates *CAMK2A* through AU‐rich element (ARE) in its 3′‐untranslated region (3′‐UTR), dual‐luciferase reporter assays were performed. The WT *CAMK2A* 3′‐UTR sequence containing predicted ARE binding sites was amplified by PCR and cloned into the psiCHECK‐2 vector (Promega) downstream of the Renilla luciferase gene. A mutant (MUT) construct with deleted or mutated ARE sequences was generated using site‐directed mutagenesis. The TNF 3′‐UTR, a known target of zinc finger protein 36 (ZFP36) family proteins, was used as a positive control. HEK293T cells or nasal epithelial cells were co‐transfected with reporter plasmids (WT or MUT CAMK2A 3′‐UTR, or TNF 3′‐UTR) together with ZFP36L1 expression vectors (shZFP36L1, oeZFP36L1 or empty vector controls) using Lipofectamine 3000 (Invitrogen). After 48 h, cells were lysed, and luciferase activities were measured using the dual‐luciferase reporter assay system (Promega) according to the manufacturer's protocol. Renilla luciferase activity was normalized to firefly luciferase activity for each sample. All experiments were performed in triplicate.

### 2.11 Validation in animal models

The expression levels of ZFP36L1 and CAMK2A were examined in the animal models described in Section 2.3 (WT‐NC, WT‐LPS, *ZFP36L1* ^KO^‐NC and *ZFP36L1* ^KO^‐LPS groups). Multiplex immunofluorescence staining was performed to visualize the spatial colocalization of *ZFP36L1* and *CAMK2A* in nasal mucosal tissues. Briefly, paraffin‐embedded tissue sections were deparaffinized, subjected to antigen retrieval, blocked with 5% BSA, and sequentially incubated with primary antibodies against ZFP36L1 (Sigma‐Aldrich, T5327) and CAMK2A (Abcam, ab22609) overnight at 4°C. After washing, sections were incubated with fluorophore‐conjugated secondary antibodies (Alexa Fluor 488 and 594, Invitrogen), and nuclei were counterstained with DAPI. Images were acquired using a confocal laser scanning microscope (Zeiss LSM 880).

### 2.12 CAMK2A rescue experiments in vitro

To validate that CAMK2A acts as a functional downstream mediator of *ZFP36L1*‐regulated ferroptosis and mitochondrial dysfunction, rescue experiments were performed by co‐depleting *ZFP36L1* and *CAMK2A*. Human nasal epithelial cells were divided into five groups: (1) MOCK (untreated control), (2) LPS (treated with 1 µg/mL LPS for 24 h), (3) LPS+vector (LPS‐treated cells transfected with empty vector), (4) LPS+sh*ZFP36L1* (LPS‐treated cells with *ZFP36L1* knockdown) and (5) LPS+shZFP36L1+shCAMK2A (LPS‐treated cells with both ZFP36L1 and CAMK2A knockdown). Cells were transfected with specific shRNA constructs or empty vectors using Lipofectamine 3000 (Invitrogen) 24 h before LPS stimulation. After 48 h of LPS treatment, cells were collected for subsequent analyses.

### 2.13 Assessment of mitochondrial morphology and function

Mitochondrial morphology was assessed by co‐staining with Mito‐Tracker Green FM (200 nM, Invitrogen) and Hoechst 33342 (10 µg/mL, Sigma‐Aldrich) for 30 min at 37°C. After washing with PBS, live cells were immediately imaged using fluorescence microscopy. Mitochondrial number, morphology and distribution were analysed using ImageJ software. Intracellular ROS levels were measured by flow cytometry using DCFH‐DA (10 µM, Sigma‐Aldrich). Cells were incubated with DCFH‐DA for 30 min at 37°C in the dark, washed twice with PBS, trypsinized and analysed on a flow cytometer (BD FACSCanto II). Mean fluorescence intensity was quantified for at least 10 000 cells per sample.

### 2.14 Clinical sample collection and analysis

To validate the *ZFP36L1–CAMK2A* regulatory axis in human CRS, nasal mucosal tissue samples were collected from patients undergoing endoscopic sinus surgery and HCs (*n*_HC = 16, *n*_CRS without polyps = 14, *n*_CRS with polyps = 32). The expression levels of ZFP36L1 and CAMK2A were specifically examined. Immunohistochemistry was performed on paraffin‐embedded tissue sections using antibodies against ZFP36L1 (Sigma‐Aldrich, T5327) and CAMK2A (Abcam, ab22609). Staining intensity was scored semi‐quantitatively by two independent pathologists blinded to clinical information, and average optical density values were calculated using Image‐Pro Plus software. Multiplex immunofluorescence staining was performed to visualize the spatial relationship between ZFP36L1 and CAMK2A in nasal epithelial cells. The colocalization patterns were analysed, and Pearson's correlation coefficients were calculated to quantify the inverse relationship between ZFP36L1 and CAMK2A expression.

### 2.15 Statistical analysis

For experimental data, comparisons between two groups were performed using Student's *t*‐test. Multiple group comparisons were analysed by one‐way analysis of variance followed by Tukey's post hoc test. Groups sharing the same lowercase letter are not significantly different (*p* > .05), whereas groups with different letters indicate statistically significant differences (*p* < .05), as determined by Tukey's post hoc test. Correlation analyses were performed using Spearman's or Pearson correlation coefficients as appropriate. All experiments included at least three biological replicates. Statistical significance was defined as *p < .05, **p < .01, ***p < .001 and ****p < .0001; ns, not significant. All tests were two‐tailed. For clinical sample analyses, multivariate linear regression was used to estimate adjusted group differences after controlling for age and gender. Effect sizes are reported as adjusted mean differences with 95% CI.

## 3 RESULTS

### 3.1 Identification of ZFP36L1 as a key gene in CRS pathogenesis

**FIGURE 1:**
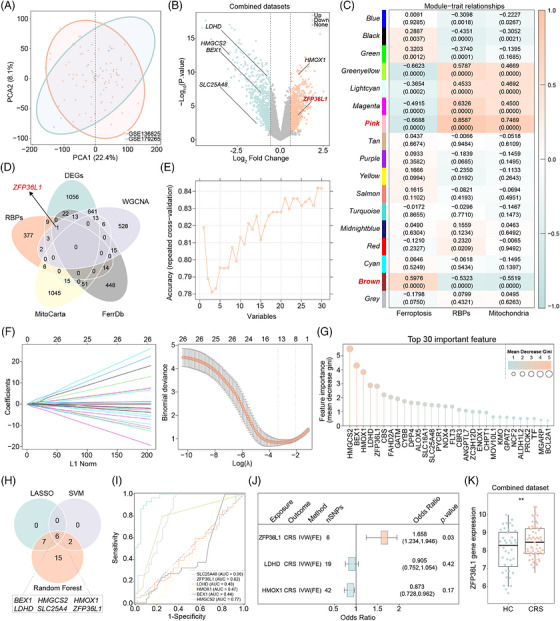
Identification of key genes of chronic rhinosinusitis (CRS). (A) Results of batch effect removal. (B) Volcano plots illustrating differentially expressed genes (DEGs) derived from comparing 59 CRS and 40 HC samples within the integrated dataset. (C) Correlation results between module and trait (RBP‐related genes [RBPRGs], ferroptosis‐related genes [FRGs] and mitochondrial‐related genes [MRGs]‐related scores). (D) Venn maps of target genes. (E–G) Results of searching characteristic genes of CRS by support vector machine recursive feature elimination (SVM‐RFE) (E), least absolute shrinkage and selection operator (LASSO) (F) and random forest (RF) (G). (H) Venn map of the characteristic genes of CRS identified by the three machine learning algorithms. (I) Receiver operating characteristic (ROC) curves of hub genes. (J) Mendelian randomization (MR) analysis. (K) Expression of key gene (ZFP36L1) in the combined dataset.

To systematically identify key genes involved in CRS pathogenesis, we integrated differential expression analysis, weighted gene co‐expression network analysis (WGCNA) and machine learning algorithms. After batch effect correction, principal component analysis demonstrated successful integration of two GEO datasets (GSE136825 and GSE179265) (Figure 1A). The quality of batch correction was assessed demonstrating that dataset‐of‐origin clustering was effectively removed (Figure S1). Differential expression analysis identified 1773 DEGs between 59 CRS and 40 HC samples, including 13 RBPRGs, 36 FRGs and 28 MRGs (Figure 1B).

WGCNA identified 15 co‐expression modules using a soft‐threshold power of 8 (Figure S2A). Module preservation analysis confirmed that key modules, including the Turquoise, Blue and Brown modules, demonstrated strong preservation (Zsummary > 10; Figure S2B). Highly similar modules were merged at a dissimilarity threshold of .25, yielding approximately eight consolidated modules (Figure S2C). Module‐trait correlation analysis revealed that the pink module exhibited the strongest positive correlations with FRGs and MRGs, whereas the brown module showed strong correlation with RBPRGs (Figure 1C, Figure S2D). By intersecting the differentially expressed RBPRGs/FRGs/MRGs with the 1222 genes from these 2 key modules, we obtained 30 candidate target genes (Figure 1D), comprising 4 RBPRGs (ENOX1, MOV10L1, ZC3H12D, ZFP36L1), 14 FRGs (ALOX5, ANGPTL7, BEX1, CBS, CYBB, DPP4, FLT3, HMOX1, NCF2, NOX4, PROK2, SLC16A1, TF, ZFP36L1) and 13 MRGs (ALDH1L2, BCL2A1, CBR3, CHPT1, FAHD2A, GATM, GPAT2, HMGCS2, KMO, LDHD, MGARP, PYCR1, SLC25A48).

Three machine learning algorithms were applied to prioritize genes with optimal predictive performance. LASSO regression selected 13 characteristic genes (Figure 1E), SVM‐RFE identified eight genes (Figure 1F), and RF ranked genes by feature importance (Figure 1G). Intersection of these three approaches yielded six hub genes: BEX1, HMGCS2, HMOX1, LDHD, SLC25A48 and ZFP36L1 (Figure 1H). ROC curve analysis demonstrated that HMGCS2, SLC25A48 and ZFP36L1 exhibited strong diagnostic performance (AUC > .7) (Figure 1I). Importantly, MR analysis revealed that only ZFP36L1 showed a significant causal relationship with CRS (Figure 1J). Expression validation confirmed significant upregulation of ZFP36L1 in CRS samples compared to controls (*p* < .001) (Figure 1K).

### 3.2 Knockout of *ZFP36L1* attenuates ferroptosis and inflammatory responses in CRS

**FIGURE 2:**
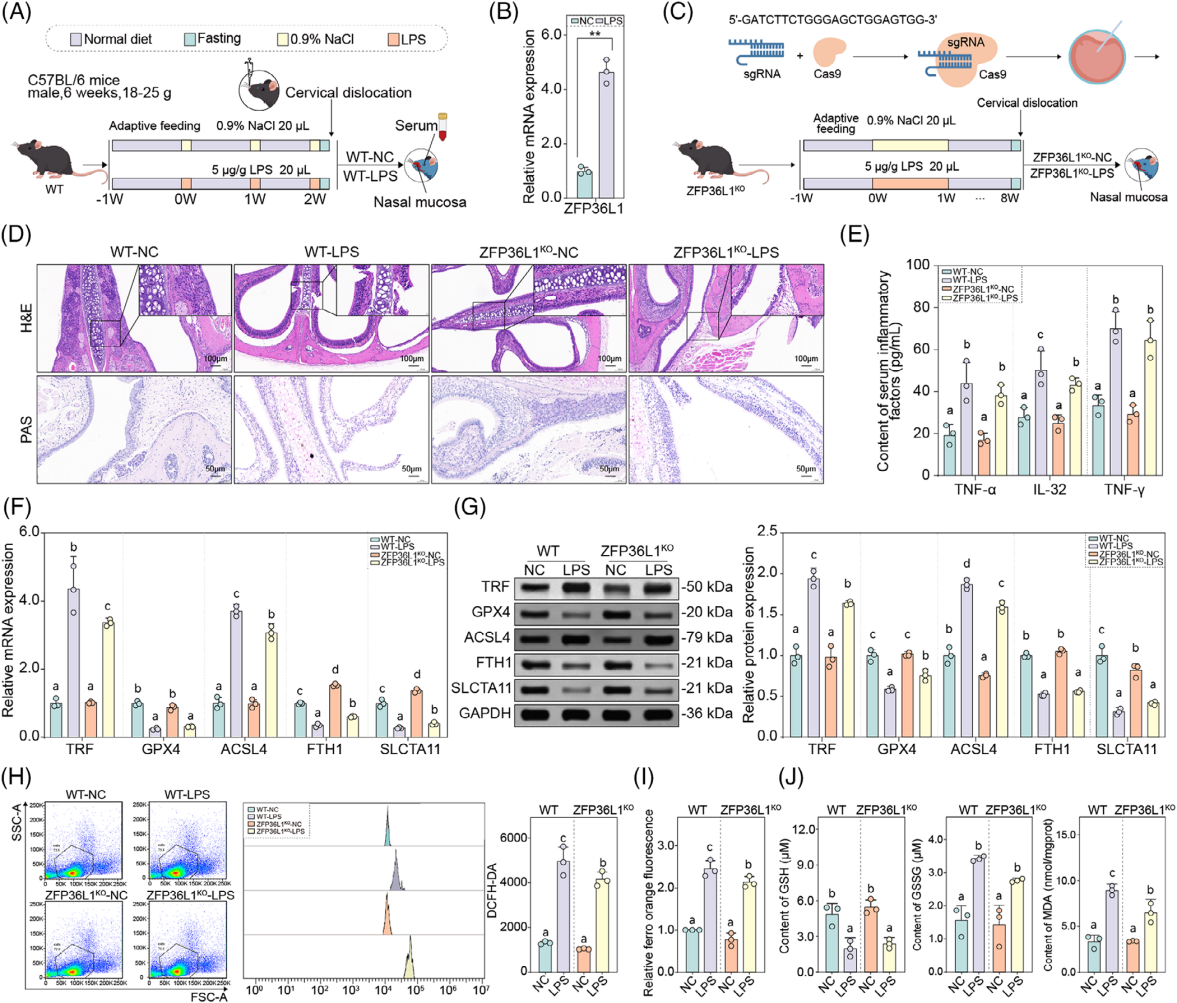
ZFP36L1 knockout attenuates ferroptosis and inflammatory responses in chronic rhinosinusitis (CRS). (A) Flow chart of CRS model establishment in this study. (B) The gene expression levels of ZFP36L1 in normal and CRS mice models (wild‐type [WT]‐NC and WT‐lipopolysaccharide [LPS]). (C) Flow chart of ZFP36L1 knockout mouse model generation using CRISPR/Cas9 technology. (D) Haematoxylin and eosin (HE) and periodic acid–Schiff (PAS) staining results of the mice sinus mucosal epithelial sections in different groups (WT‐NC, WT‐LPS, ZFP36L1^KO^‐NC and ZFP36L1^KO^‐LPS). Scale bars: 100 µm for HE staining, 50 µm for PAS staining. (E) Content of serum inflammatory factors (TNF‐α, TNF‐γ and IL‐32) in different groups. (F) The gene expression levels of ferroptosis‐related proteins (TRF, glutathione peroxidase 4 [*GPX4*], acyl‐CoA synthetase long‐chain family member 4 [*ACSL4*], ferritin heavy chain [*FTH1*] and solute carrier family 7 member 11 [*SLC7A11*]) in nasal mucosal tissue. (G) The protein expression levels of ferroptosis‐related proteins in different groups. (H) Flow cytometry analysis of intracellular reactive oxygen species (ROS) levels detected by (I) 2′,7′‐dichlorodihydrofluorescein diacetate (*DCFH‐DA*) staining in different groups. (J) Quantification of glutathione (GSH), GSSG and malondialdehyde (MDA) levels in different groups. Different letters indicate significant differences between groups (*p* < .05). Data are presented as mean ± SD (*n* ≥ 3).

To investigate the functional role of *ZFP36L1* in CRS pathogenesis, we first established a CRS model in mice using LPS nasal instillation (Figure 2A) and found that ZFP36L1 was significantly upregulated in the WT‐LPS group compared to the WT‐NC group (Figure 2B). Subsequently, *ZFP36L1* knockout mice models were constructed for further functional analyses (Figure 2C). HE staining results showed that the sinus mucosal epithelial structure of mice in the WT‐NC and *Z* *FP36L1* ^KO^‐NC groups was well‐preserved with no obvious abnormalities, minimal inflammatory cell infiltration and gland hyperplasia, whereas in WT‐LPS and *ZFP36L1* ^KO^‐LPS groups, the epithelial cells were disorganized with obvious inflammatory cell infiltration. Notably, *ZFP36L1* ^KO^‐LPS mice showed less severe inflammatory damage compared to WT‐LPS mice. Similarly, PAS staining results showed that there was less purple sediment in the sinus tissue of mice in WT‐NC and *ZFP36L1* ^KO^‐NC groups with intact mucosal structure and no inflammatory reaction, whereas in WT‐LPS and *ZFP36L1* ^KO^‐NC groups, the sinus tissue showed increased purple sediment, with less accumulation in the knockout group (Figure 2D, Figure S3A,B). Serum inflammatory factors (TNF‐α, TNF‐γ and IL‐32) were significantly increased in WT‐LPS group compared with the WT‐NC group (*p* < .05). No significant differences were observed between WT‐NC and *ZFP36L1* ^KO^‐NC groups, indicating that ZFP36L1 knockout alone does not affect basal inflammatory status (Figure 2E).

Subsequently, we analysed the effect of ZFP36L1 on ferroptosis regulation. qRT‐PCR and WB results showed that the expressions of pro‐ferroptotic markers *TRF* and *ACSL4* were significantly increased (*p* < .05), whereas anti‐ferroptotic proteins GPX4, FTH1 and SLC7A11 were significantly decreased in WT‐LPS group (*p* < .05). Importantly, knockout of ZFP36L1 substantially reversed these ferroptotic changes (Figure 2F,G). Compared with WT‐NC and *ZFP36L1* ^KO^‐NC groups, the ROS content and Fe^2+^ content were significantly increased in WT‐LPS group and were significantly reversed in *ZFP36L1* ^KO^‐LPS group (*p* < .05) (Figure 2H,I). Furthermore, the GSH level was decreased, whereas GSSG and MDA levels were significantly increased in WT‐LPS group and were significantly reversed in *ZFP36L1* ^KO^‐LPS group (*p* < .05), with no significant differences between WT‐NC and *ZFP36L1* ^KO^‐NC groups (*p* > .05) (Figure 2J). Collectively, these results demonstrated that knockout of *ZFP36L1* effectively attenuated the ferroptotic phenotype of CRS, restoring the balance between pro‐ and anti‐ferroptotic signalling and normalizing cellular redox homeostasis.

### 3.3 The effects of ZFP36L1 knockout on the mitochondrial quality control system in CRS

**FIGURE 3:**
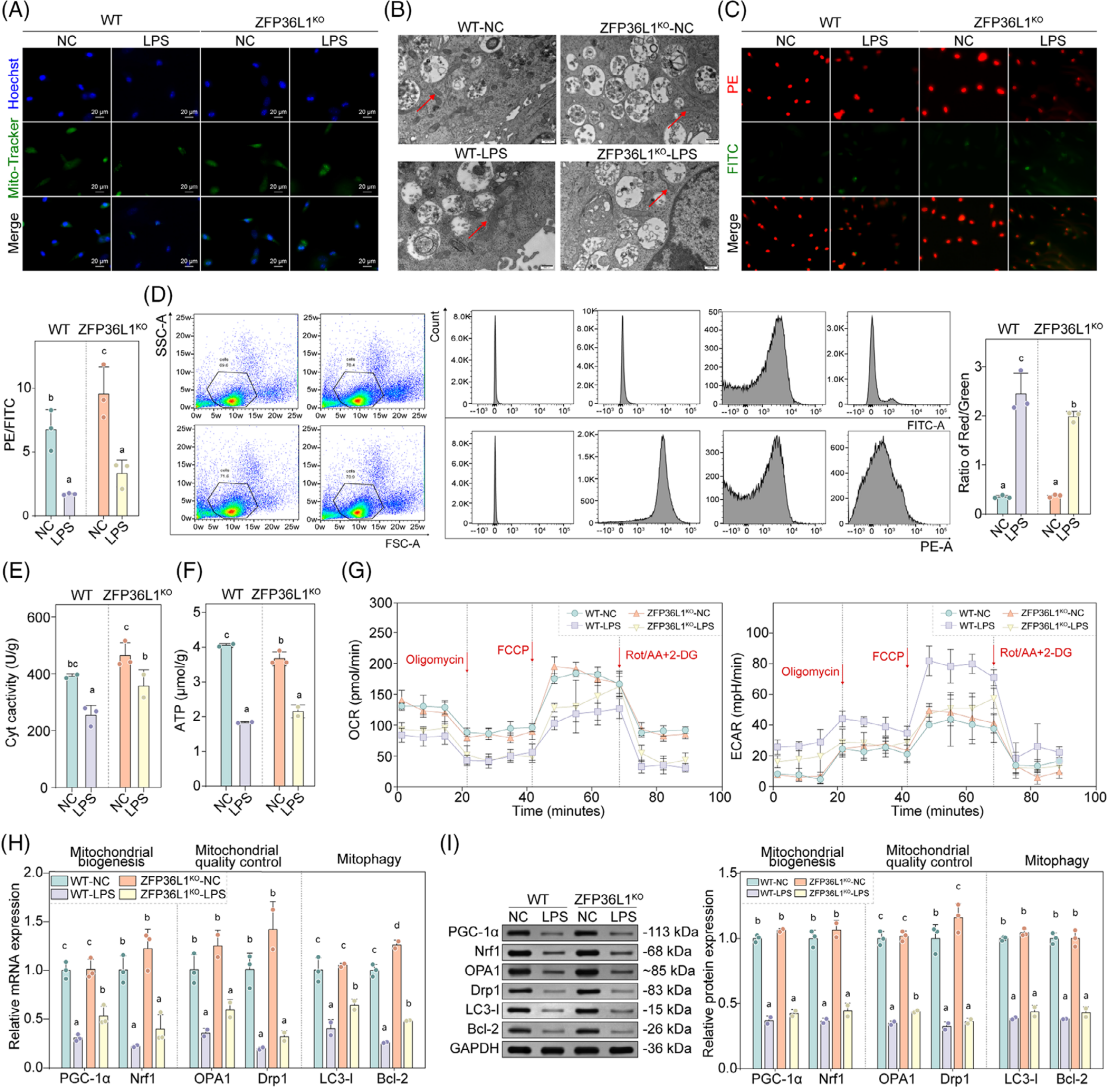
The effects of ZFP36L1 knockout on the mitochondrial quality control system in chronic rhinosinusitis (CRS). (A) Co‐localization staining of Mito‐Tracker (green) and Hoechst 33342 (blue) showing mitochondrial morphology and abundance in nasal mucosal tissues. (B) TEM analysis of mitochondrial ultrastructure. (C) Flow cytometry analysis of mitochondrial membrane potential showing PE/FITC fluorescence ratios and representative scatter plots. (D) Quantification of mitochondrial membrane potential as red/green fluorescence intensity ratio from JC‐1 staining. (E) The DCytaa3 activity level in different groups. (F) ATP levels in different groups. (G) Seahorse metabolic analysis showing change of oxygen consumption rate (OCR) and extracellular acidification rate (ECAR) in different groups. (H) mRNA expression levels of the biomarkers of mitochondrial biogenesis markers (PGC‐1α and Nrf1), mitochondrial quality control (OPA1 and Drp1) and mitophagy markers (LC3I and Bcl‐2) in different groups. (I) Protein expression levels of the biomarkers of mitochondrial biogenesis, mitochondrial quality control and mitophagy in different groups. Different letters indicate significant differences between groups (*p* < .05); data are presented as mean ± SD (*n* ≥ 3). ATP, adenosine triphosphate; LPS, lipopolysaccharide; OCR, oxygen consumption rate; WT, wild‐type.

*ZFP36L1* knockout significantly attenuated LPS‐induced mitochondrial dysfunction. Laser confocal microscopy showed that mitochondria in WT‐NC and *ZFP36L1* ^KO^‐NC groups were moderate, roughly round or oval, with intact morphology, whereas the number of mitochondria in WT‐LPS group was significantly reduced, with swollen morphology and some fragmented mitochondria. Notably, mitochondrial numbers in *ZFP36L1* ^KO^‐LPS mice were also reduced but with less severe swelling and fragmentation compared to WT‐LPS (Figure 3A, Figure S4A). TEM results confirmed this pattern: Mitochondria in WT‐NC and ZFP36L1^KO^‐NC groups were oval or spindle‐shaped with clear internal structure, complete inner and outer membrane and better mitochondrial quality. Mitochondria in both WT‐LPS and ZFP36L1ZFP36L1^KO^‐LPS groups were swollen and dilated with disorganized internal structure and vacuoles or electron‐dense material deposition, though structural disruption was less severe in the ZFP36L1^KO^‐LPS group (Figure 3B, Figure S4B). The mitochondrial membrane potential (ratio of PE/FITC and ratio of red/green) was higher in WT‐NC and ZFP36L1^KO^‐NC groups, whereas it was significantly lower in WT‐LPS and ZFP36L1^KO^‐LPS groups (Figure 3C,D, Figure S4C). DCytaa3 activity and ATP levels were significantly reduced in WT‐LPS and ZFP36L1^KO^‐LPS groups compared with WT‐NC and ZFP36L1^KO^‐NC groups (Figure 3E,F). Seahorse analysis showed that oxygen consumption rate (OCR) and extracellular acidification rate (ECAR) in ZFP36L1^KO^‐NC group were similar to WT‐NC, whereas those in WT‐LPS and ZFP36L1^KO^‐LPS groups showed similar patterns (Figure 3G). mRNA and protein expression levels of biomarkers for mitochondrial biogenesis (PGC‐1α and Nrf1), mitochondrial quality control (OPA1 and Drp1) and mitophagy (LC3I and Bcl‐2) were significantly decreased in WT‐LPS group, and only OPA1 and LC3I were significantly reversed in ZFP36L1^KO^‐LPS group (Figure 3H,I), confirming the important impact of ZFP36L1 knockout on the mitochondrial quality control system in CRS.

### 3.4 Ferroptosis mediates ZFP36L1‐regulated mitochondrial dysfunction in CRS

**FIGURE 4:**
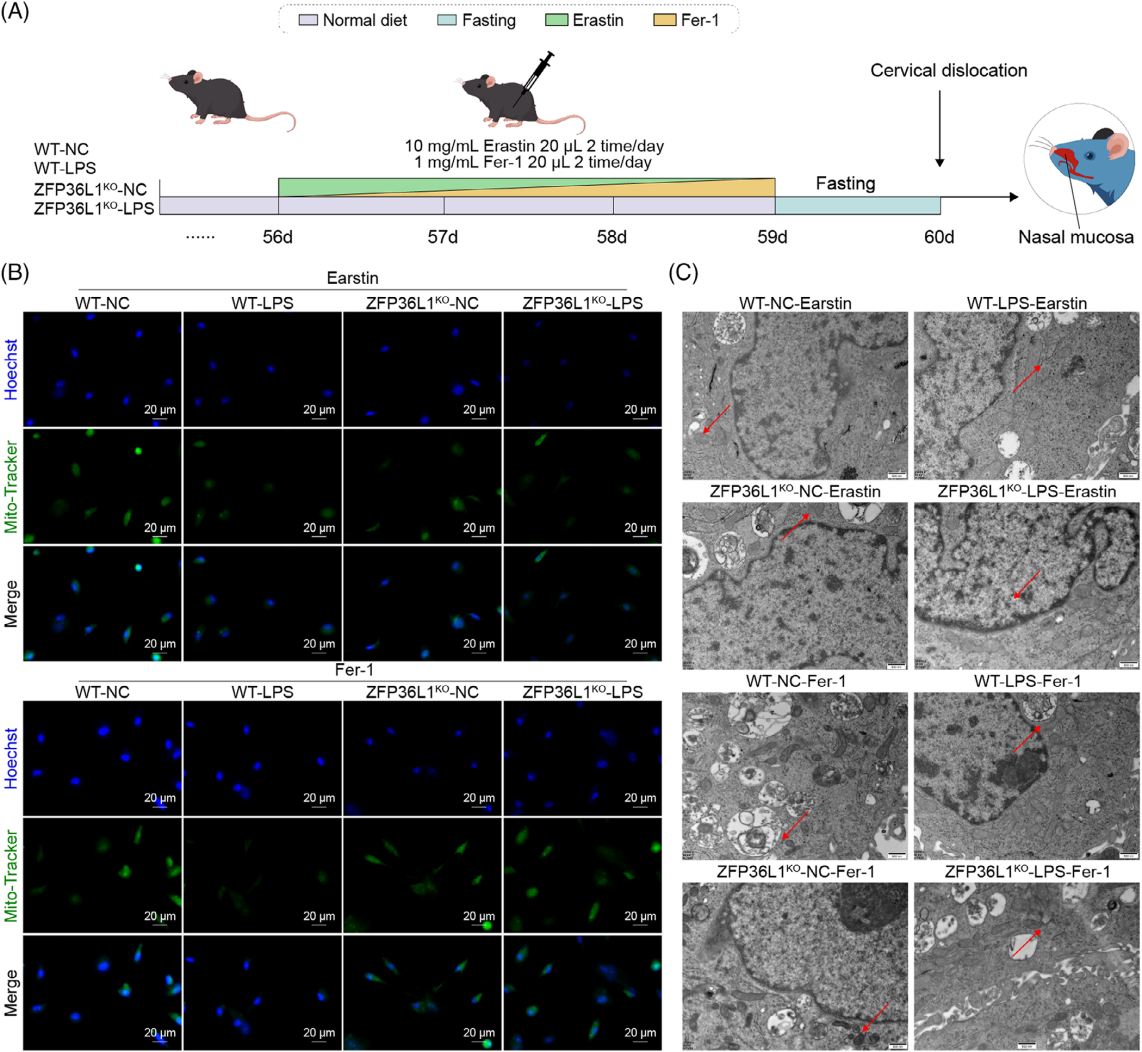
The association of ferroptosis and mitochondrial quality control system in chronic rhinosinusitis (CRS). (A) Flow chart showing ferroptosis modulation in wild‐type (WT) and *ZFP36L1* ^KO^ mice with lipopolysaccharide (LPS)‐induced CRS, treated with ferroptosis inducer erastin or inhibitor Fer‐1. (B) Mito‐Tracker (green) and Hoechst 33342 (blue) colocalization showing mitochondrial morphology and abundance across all treatment groups. (C) TEM analysis of mitochondrial ultrastructure demonstrating changes in morphology and internal organization under ferroptosis modulation.

**FIGURE 5:**
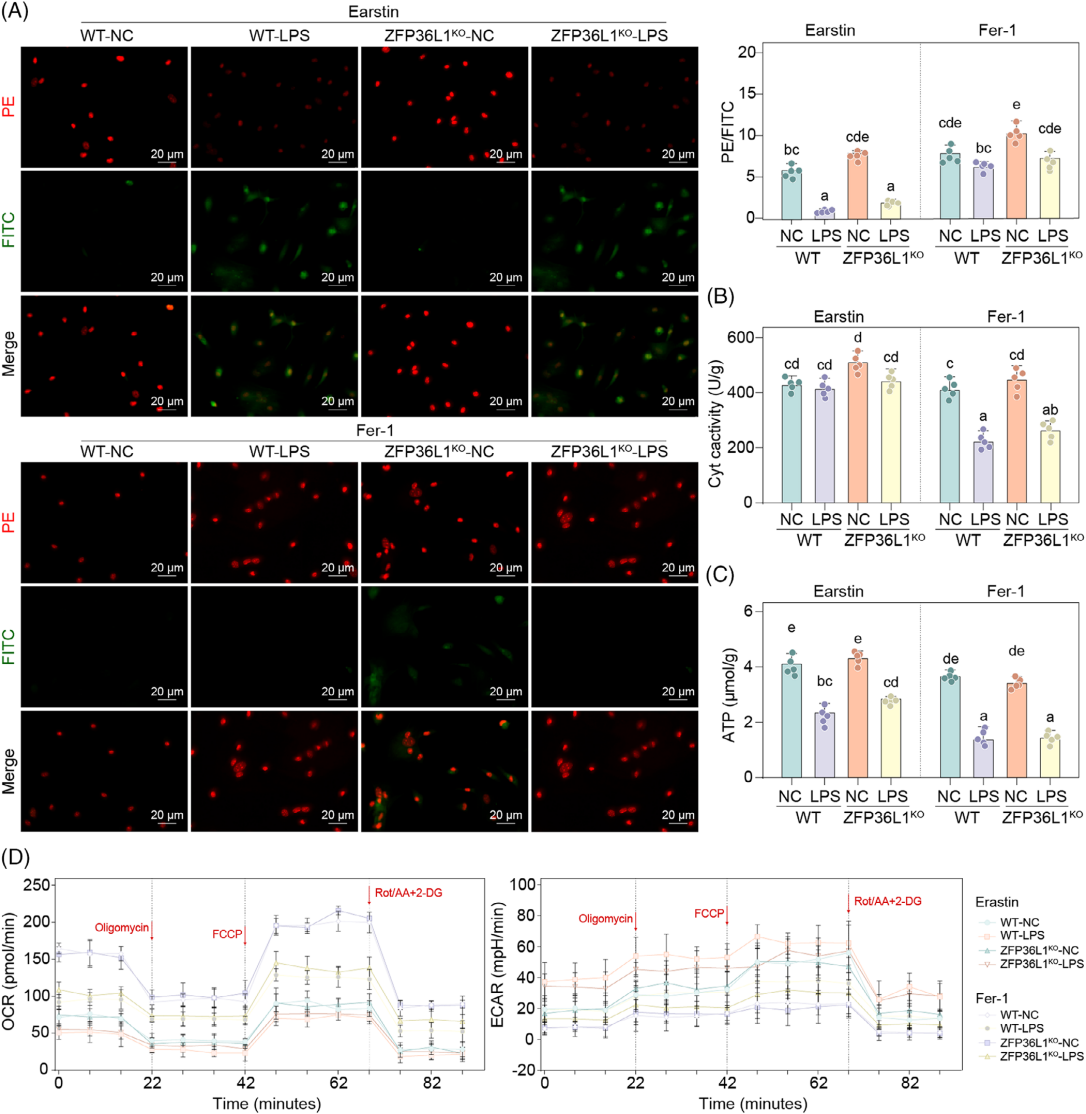
The association of ferroptosis and mitochondrial quality control system in chronic rhinosinusitis (CRS). (A) Mitochondrial membrane potential measured as red/green fluorescence intensity ratio from JC‐1 staining across all treatment groups. (B) The DCytaa3 activity level in different groups. (C) The ATP levels in different groups. (D) Seahorse metabolic analysis showing oxygen consumption rate (OCR) and extracellular acidification rate (ECAR) under ferroptosis modulation. Different letters indicate significant differences between groups (*p* < .05); data are presented as mean ± SD (*n* ≥ 3). ATP, adenosine triphosphate; LPS, lipopolysaccharide; WT, wild‐type.

To determine whether ferroptosis mediates *ZFP36L1*‐regulated mitochondrial dysfunction, we treated CRS models with erastin or Fer‐1 to modulate ferroptosis status and assess downstream effects on mitochondrial quality control (Figure 4A). Mito‐Tracker and Hoechst 33342 colocalization showed minimal mitochondrial abnormalities in WT‐NC and *ZFP36L1* ^KO^‐NC groups. In contrast, WT‐LPS, WT‐LPS+erastin and *ZFP36L1* ^KO^‐LPS+erastin groups exhibited markedly reduced mitochondrial numbers with widespread swelling and fragmentation. Notably, Fer‐1 treatment significantly increased mitochondrial number with only minimal swelling in both WT‐LPS+Fer‐1 and *ZFP36L1* ^KO^‐LPS+Fer‐1 groups compared to erastin‐treated counterparts (Figure 4B). TEM confirmed this pattern: Mitochondria in WT‐NC, *ZFP36L1* ^KO^‐NC, WT‐LPS+Fer‐1 and *ZFP36L1* ^KO^‐LPS+Fer‐1 groups maintained oval or spindle‐shaped morphology with clear internal structure and intact inner/outer membranes. Conversely, WT‐LPS+erastin and *ZFP36L1* ^KO^‐LPS+erastin groups exhibited severe mitochondrial swelling with disorganized internal structure and extensive vacuole/electron‐dense material deposition (Figure 4C). Mitochondrial membrane potential was markedly decreased in erastin‐treated groups but significantly restored by Fer‐1 treatment (Figure 5A). DCytaa3 activity and ATP levels followed a similar pattern: Erastin treatment decreased both parameters compared to LPS alone, whereas Fer‐1 significantly increased both parameters (Figure 5B,C). Seahorse metabolic analysis revealed that erastin treatment suppressed OCR and ECAR, whereas Fer‐1 treatment restored both parameters (Figure 5D). Collectively, these findings demonstrate that ferroptosis is a key mechanism through which ZFP36L1 dysfunction leads to mitochondrial dysfunction in CRS.

### 3.5 ZFP36L1 directly targets CAMK2A mRNA for degradation in CRS

**FIGURE 6:**
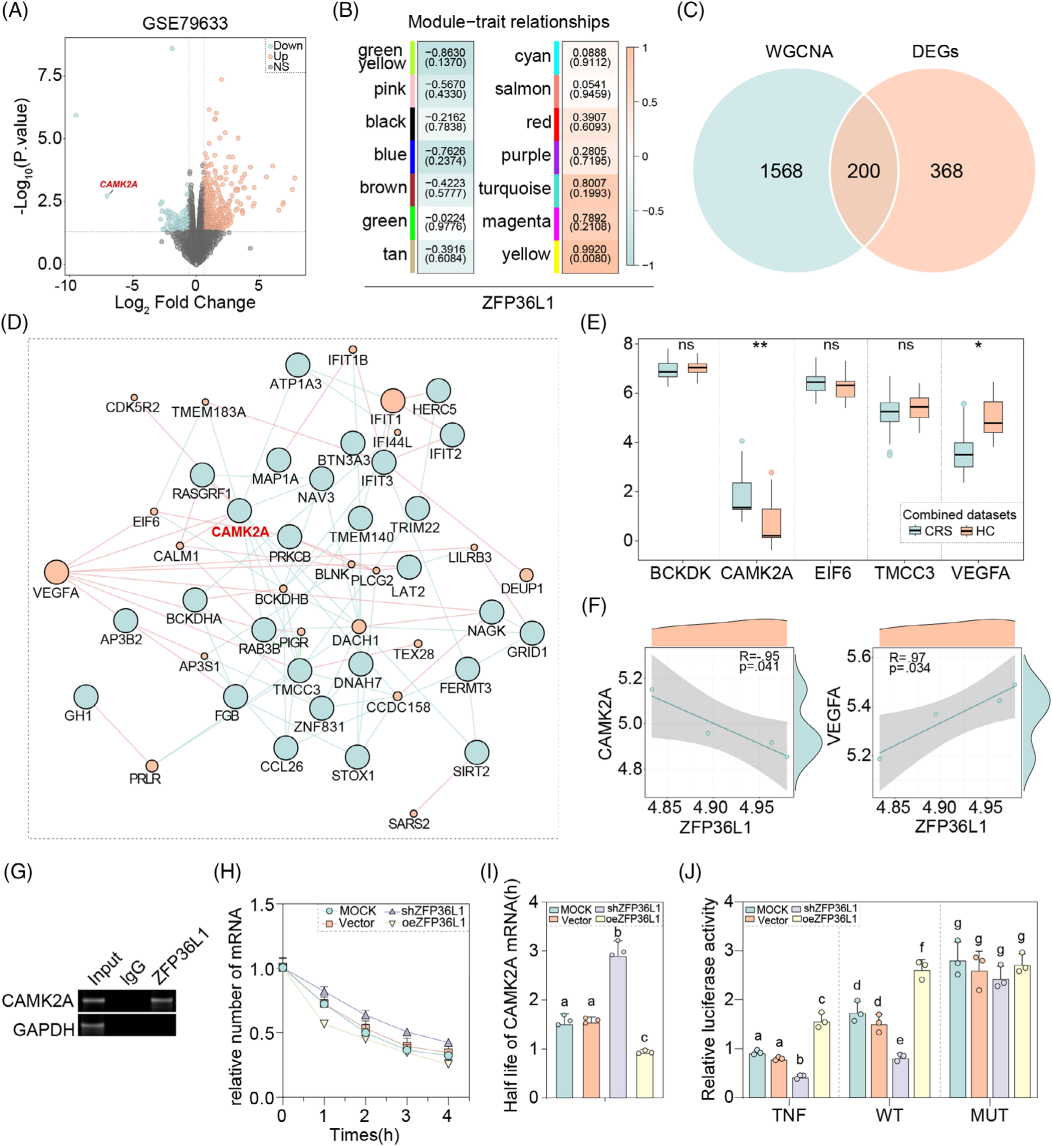
The regulatory mechanism of *ZFP36L1* and *CAMK2A* in chronic rhinosinusitis (CRS). (A) Volcano plots depicting the differentially expressed genes (DEGs) between samples with *ZFP36L1* overexpression and those with normal expression levels. (B) Correlation analysis between the module and the trait, defined as *ZFP36L1* expression. (C) The Venn maps of target genes. (D) The PPI network of target genes. (E) The expression of core genes (top 5 genes in protein–protein interaction [PPI] network) between 59 CRS and 40 HC samples in combined dataset. (F) Correlation analysis between *ZFP36L1* and core genes. (G) RNA immunoprecipitation (RIP) assay confirming direct binding of *ZFP36L1* to *CAMK2A*. (H and I) mRNA stability assay following actinomycin D treatment (5 µg/mL) to determine half‐life of *CAMK2*A mRNA. (J) Dual‐luciferase reporter assay in HEK293T cells co‐transfected with *ZFP36L1*‐modulating constructs and reporters containing wild‐type (WT) or AU‐rich element (ARE) MUT 3′‐untranslated region (3′‐UTR) of *CAMK2A* or TNF (positive control); relative luciferase activity normalized to Renilla. Different letters indicate significant differences between groups (*p* < .05); data are presented as mean ± SD (*n* ≥ 3).

To identify the direct molecular targets through which *ZFP36L1* mediates ferroptosis and mitochondrial dysfunction, we analysed ZFP36L1 overexpression‐related gene expression data (GSE79633) combined with WGCNA and machine learning approaches. Module‐trait correlation analysis revealed that genes associated with *ZFP36L1* expression clustered in specific modules, with subsequent PPI network analysis identifying a core regulatory network centred on *ZFP36L1* and its direct interacting partners. Among the candidate downstream targets, CAMK2A emerged as a high‐confidence target showing strong correlation with *ZFP36L1* expression in the combined CRS datasets. Volcano plot analysis highlighted CAMK2A as a significantly downregulated gene in CRS samples compared to HCs (Figure 6A). WGCNA module‐trait correlation analysis demonstrated that CAMK2A clustered within modules that were significantly associated with *ZFP36L1* expression pattern (Figure 6B). Venn diagram analysis confirmed that CAMK2A was identified as a target gene by the intersection of WGCNA‐identified module genes and DEGs (Figure 6C). PPI network analysis further validated *CAMK2A* as a core node within the ZFP36L1‐centred regulatory network, with multiple direct PPIs evident (Figure 6D). Expression analysis in the combined CRS datasets showed that *CAMK2A* levels were significantly downregulated in CRS compared to HCs (*p* < .01), whereas other candidate targets (*BCKDK, EIF6, TMCC3* and *VEGFA)* showed no significant differences or inconsistent patterns (Figure 6E). To determine whether *CAMK2A* is a direct target of *ZFP36L1* binding, we performed correlation analysis in the combined dataset. Spearman's correlation analysis revealed a significant inverse correlation between *ZFP36L1* and *CAMK2A* expression (Figure 6F, left panel). In contrast, *ZFP36L1* showed positive correlation with VEGFA (Figure 6F, right panel). To verify direct binding between ZFP36L1 protein and *CAMK2A* mRNA, RIP assays were performed with anti‐ZFP36L1 antibodies. *CAMK2A* mRNA was specifically enriched in ZFP36L1 immunoprecipitates compared to IgG control, with GAPDH mRNA showing minimal enrichment, confirming direct ZFP36L1–CAMK2A interaction (Figure 6G). To examine whether ZFP36L1 regulates *CAMK2A* mRNA stability, we conducted mRNA decay assays following actinomycin D treatment. *ZFP36L1* knockdown (shZFP36L1) significantly extended CAMK2A mRNA half‐life compared to control and vector groups, whereas *ZFP36L1 o*verexpression (oe*ZFP36L1*) markedly reduced *CAMK2A* mRNA half‐life (Figure 6H,I), demonstrating that *ZFP36L1* directly destabilizes *CAMK2A* mRNA. To determine whether *ZFP36L1* regulates *CAMK2A* through ARE in its 3′‐UTR, we constructed luciferase reporter plasmids containing either WT or ARE‐mutated (MUT) *CAMK2A* 3′‐UTR sequences. TNF 3′‐UTR, a well‐characterized target of ZFP36 family proteins, served as a positive control. Dual‐luciferase assays showed that ZFP36L1 knockdown significantly increased luciferase activity driven by both TNF 3′‐UTR and WT *CAMK2A* 3′‐UTR, whereas ZFP36L1 overexpression suppressed their activity (Figure 6J). Importantly, mutation of the ARE sequences abolished these regulatory effects, with no significant differences observed between experimental groups using the MUT *CAMK2A* 3′‐UTR construct (*p* > .05). These results confirm that *ZFP36L1 r*egulates *CAMK2A* expression through direct binding to AREs in its 3′‐UTR. Collectively, these findings demonstrate that *ZFP36L1* directly binds to *CAMK2A* mRNA and promotes its degradation through ARE‐mediated mechanisms, establishing *CAMK2A* as a critical downstream target of *ZFP36L1* in CRS.

### 3.6 CAMK2A knockdown reverses the protective effects of ZFP36L1 depletion on ferroptosis and mitochondrial dysfunction

**FIGURE 7:**
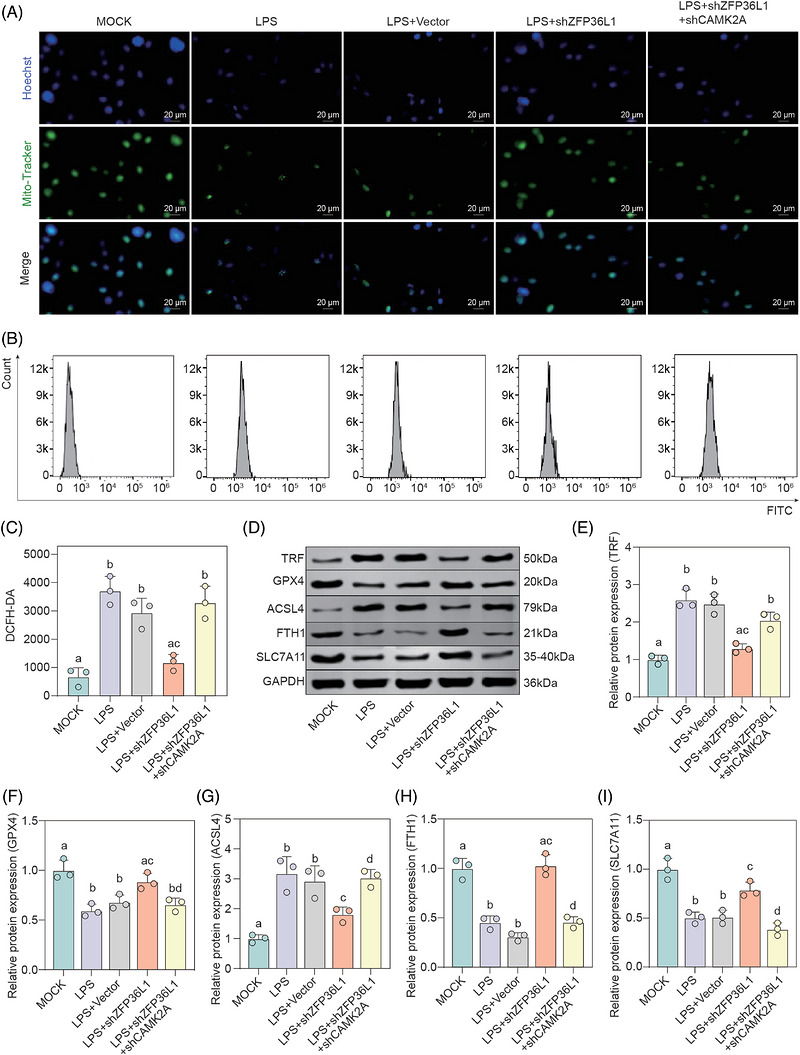
*CAMK2A* knockdown reverses the protective effects of *ZFP36L1* depletion on ferroptosis and mitochondrial dysfunction. (A) Mitochondrial morphology visualized by Mito‐Tracker (green) and Hoechst 33342 (blue) staining in nasal epithelial cells from different experimental groups (MOCK, lipopolysaccharide (LPS), LPS+vector, LPS+sh*ZFP36L1* and LPS+sh*ZFP36L1*+sh*CAMK2A*). Scale bar: 20 µm. (B) Flow cytometry analysis of intracellular reactive oxygen species (ROS) levels detected by 2′,7′‐dichlorodihydrofluorescein diacetate (DCFH‐DA) staining in five experimental groups. (C) Quantification of ROS fluorescence intensity (DCFH‐DA) from panel B. Different letters indicate significant differences between groups (*p* < .05). Data are presented as mean ± SD (*n* = 3). (D) Western blot analysis of ferroptosis‐related proteins (TRF, glutathione peroxidase 4 [GPX4], acyl‐CoA synthetase long‐chain family member 4 [ACSL4], ferritin heavy chain [FTH1] and solute carrier family 7 member 11 [SLC7A11]) in five experimental groups. GAPDH was used as a loading control. (E–I) Quantification of protein expression levels from panel D. (E) TRF; (F) GPX4; (G) ACSL4; (H) FTH1; (I) SLC7A11. Different letters indicate significant differences between groups (*p* < .05). Data are presented as mean ± SD (*n* = 3).

To validate that *CAMK2A* is a functional downstream mediator of *ZFP36L1*‐regulated ferroptosis, we performed rescue experiments by co‐depleting *ZFP36L1* and *CAMK2A* in LPS‐stimulated nasal epithelial cells. Mito‐Tracker and Hoechst 33342 colocalization analysis showed robust mitochondrial abundance and normal morphology in MOCK and LPS+vector control groups, whereas LPS stimulation dramatically reduced mitochondrial numbers with swollen and fragmented morphology. *ZFP36L1* knockdown (LPS+sh*ZFP36L1*) significantly restored mitochondrial numbers and morphology compared to LPS+vector controls. Notably, *CAMK2A* knockdown in the context of *ZFP36L1* depletion (LPS+shZFP36L1+shCAMK2A) substantially reversed the protective phenotype, restoring the pathological mitochondrial morphology characteristic of LPS‐stimulated cells (Figure 7A). Flow cytometry analysis of intracellular ROS content revealed minimal ROS accumulation in MOCK and LPS+vector groups, with dramatic ROS elevation following LPS stimulation. *ZFP36L1* knockdown significantly attenuated LPS‐induced ROS accumulation, whereas *CAMK2A* depletion in shZFP36L1 cells reversed this ROS‐suppressive effect, restoring ROS levels to near‐LPS baseline (Figure 7B,C). Western blot analysis confirmed that ferroptosis‐related proteins were profoundly dysregulated in LPS‐stimulated cells: pro‐ferroptotic markers TRF and *ACSL4* were significantly elevated, whereas anti‐ferroptotic proteins GPX4, FTH1 and SLC7A11 were substantially decreased. *ZFP36L1* knockdown substantially reversed these ferroptotic signatures, restoring the balance towards an anti‐ferroptotic state. Critically, CAMK2A knockdown reversed the protective effects of ZFP36L1 depletion: in LPS+shZFP36L1+shCAMK2A cells, TRF and ACSL4 expression increased, whereas GPX4, FTH1 and SLC7A11 returned to LPS levels, demonstrating that CAMK2A depletion abolishes the ferroptosis‐protective phenotype conferred by ZFP36L1 depletion (Figure 7D–I). To further validate the specificity of the ZFP36L1–CAMK2A regulatory axis, we generated CAMK2A knockout nasal epithelial cells using CRISPR/Cas9. Western blot confirmed successful ablation of CAMK2A protein expression (Figure S5A). Notably, CAMK2A‐KO cells exhibited significantly elevated levels of pro‐ferroptotic markers (ACSL4) and reduced anti‐ferroptotic proteins (GPX4, FTH1 and SLC7A11), accompanied by increased ROS accumulation and decreased mitochondrial membrane potential, even in the absence of LPS stimulation (Figure S5B–D). These results demonstrate that CAMK2A loss‐of‐function is sufficient to induce the ferroptosis and mitochondrial dysfunction phenotype observed in CRS. Collectively, these experiments definitively establish that CAMK2A is both necessary and sufficient as a functional downstream target through which ZFP36L1 regulates ferroptosis and mitochondrial quality control in CRS pathogenesis.

### 3.7 Clinical validation of the ZFP36L1–CAMK2A axis in CRS patients

**FIGURE 8:**
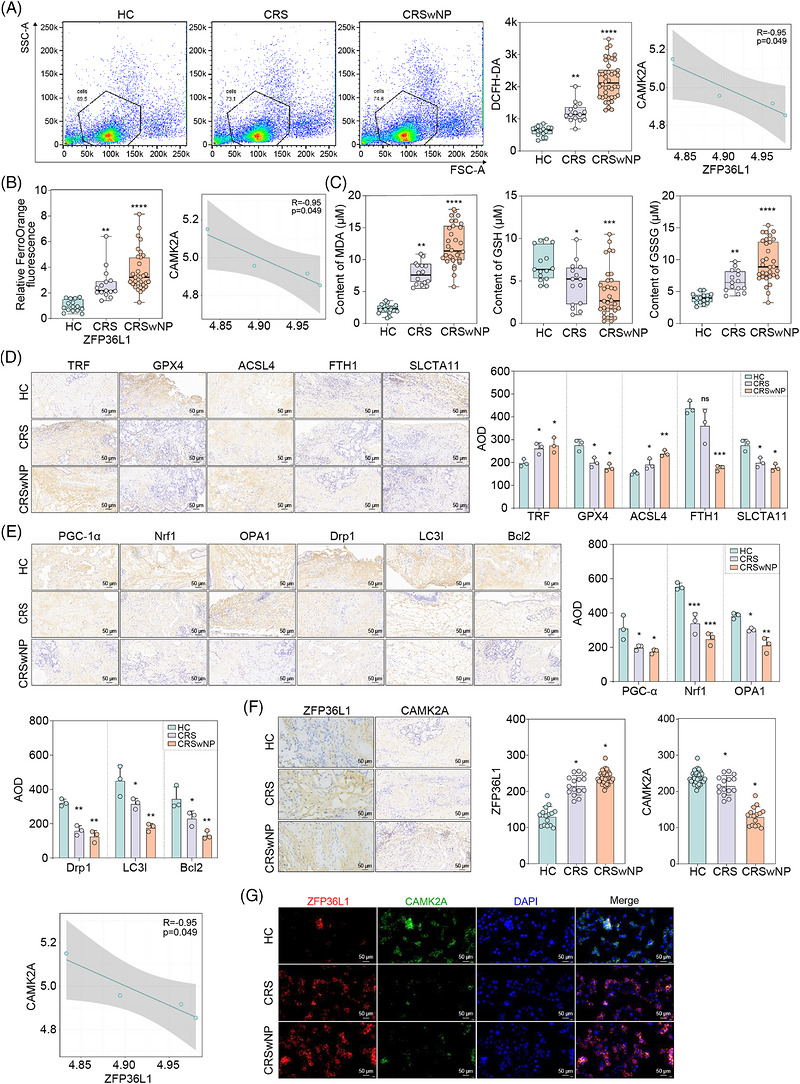
The function and regulatory mechanism of ZFP36L1 in clinical chronic rhinosinusitis (CRS) samples. (A and B) Progressive elevation of reactive oxygen species (ROS) and Fe^2+^ from healthy control (HC) to CRS to CRS with nasal polyp (CRSwNP), with positive correlation between ZFP36L1 and ROS. (C) glutathione (GSH) depletion and accumulation of GSSG and malondialdehyde (MDA) in disease groups. (D) Immunohistochemistry showing upregulation of pro‐ferroptotic markers (TRF, acyl‐CoA synthetase long‐chain family member 4 [ACSL4]) and downregulation of anti‐ferroptotic proteins (glutathione peroxidase 4 [GPX4], ferritin heavy chain [FTH1] and solute carrier family 7 member 11 [SLC7A11]). (E) Progressive dysregulation of mitochondrial biogenesis (PGC‐1α, Nrf1), quality control (OPA1, Drp1) and mitophagy markers (LC3I, Bcl2) correlating with disease severity. (F) Quantitative immunohistochemistry demonstrating inverse relationship between ZFP36L1 and CAMK2A levels. (G) Multiplex immunofluorescence revealing spatial colocalization of ZFP36L1 (red) and CAMK2A (green) in HC, CRS and CRSwNP samples. Significant differences between groups: *p* < .05; data are mean ± SD.

To translate our mechanistic findings to human disease, nasal mucosal samples were collected from HC (*n* = 16), CRS (*n* = 14) and CRSwNP patients (*n* = 32) (Table 1). Ferroptosis was substantially elevated in CRS tissues, evidenced by ROS and Fe^2+^ levels that increased progressively from HC through CRS to CRSwNP (*p* < .01 to *p* < .0001) (Figure 8A,B). Redox imbalance was pronounced, with decreased GSH but markedly elevated GSSG and MDA in disease groups (Figure 8C). Consistent with this pro‐ferroptotic state, pro‐ferroptotic markers *TRF* and *ACSL4* were upregulated, whereas anti‐ferroptotic proteins GPX4, FTH1 and SLC7A11 were suppressed in CRS/CRSwNP (Figure 8D). Mitochondrial dysfunction was evident from dysregulated biogenesis and quality control markers, with OPA1, LC3I and Bcl2 displaying disease‐severity‐dependent progressive changes (Figure 8E). Critically, an inverse expression relationship between *ZFP36L1* and *CAMK2A* paralleled disease progression. Quantitative analysis revealed stepwise increase of *ZFP36L1* from HC through CRS to CRSwNP, accompanied by reciprocal decline of *CAMK2A* from HC to CRS to CRSwNP. Correlation analysis confirmed significant negative association between these proteins (Table 2, Figure 8F). Spatial colocalization by multiplex immunofluorescence showed that high ZFP36L1 expression directly corresponded with suppressed CAMK2A in epithelial cells across all groups (Figure 8G). These clinical data validate the *ZFP36L1–CAMK2A* axis as a central mediator of ferroptosis and mitochondrial dysfunction in human CRS, positioning *ZFP36L1* as a compelling therapeutic target. To enhance statistical transparency, we performed confounder‐adjusted analyses. After adjusting for age and gender, ZFP36L1 expression remained significantly elevated across all pairwise comparisons (CRS vs. HC, CRSwNP vs. HC, CRSwNP vs. CRS; all adjusted *p* < .001), whereas *CAMK2A* showed consistent inverse downregulation (all adjusted *p* < .001) (Figure S6A,B). Subgroup analysis by disease severity (endoscopic score) revealed a dose‐dependent pattern: ZFP36L1 expression increased progressively from mild to moderate to severe subgroups, whereas *CAMK2A* expression decreased correspondingly (Figure S6C). These severity‐stratified results further support the pathological relevance of the *ZFP36L1–CAMK2A* axis in CRS progression.

**TABLE 1:** The characteristics of different groups.

	Total	HC	CRS	CRSsNP	*p*
*N*	62	16	14	32	
Gender, *n* (%)					.204
Male	38 (62.3)	11 (73.3)	6 (42.9)	21 (65.6)	
Female	24 (37.7)	5 (26.7)	8 (57.1)	11 (34.4)	
Age	45.7 ± 17.2	39.8 ± 14.9	46.9 ± 14.4	47.9 ± 18.9	.308
Left endoscopic score	3.0 (2.0, 5.0)	1.0 (1.0, 2.0)	2.0 (2.0, 3.0)	5.0 (3.0, 5.0)	<.001
Polyp	.0 (.0, 2.0)	.0 (.0, .0)	.0 (.0, .0)	2.0 (1.0, 2.0)	<.001
Oedema	1.0 (1.0, 1.0)	1.0 (.0, 1.0)	1.0 (1.0, 1.0)	1.0 (1.0, 1.0)	.168
Nasal leakage	1.0 (1.0, 2.0)	1.0 (.0, 1.0)	1.0 (1.0, 2.0)	2.0 (1.0, 2.0)	.001
Right endoscopic score	3.0 (2.0, 5.0)	1.0 (1.0, 2.0)	3.0 (2.0, 3.0)	4.0 (3.0, 5.0)	<.001
Polyp	.0 (.0, 2.0)	.0 (.0, .0)	.0 (.0, .0)	2.0 (.8, 2.0)	<.001
Oedema	1.0 (1.0, 1.0)	1.0 (.0, 1.0)	1.0 (1.0, 1.0)	1.0 (1.0, 1.0)	.046
Nasal leakage	1.0 (1.0, 2.0)	1.0 (.0, 1.0)	2.0 (1.0, 2.0)	2.0 (1.0, 2.0)	.003
Left‐sided CT score	6.0 (3.5, 10.0)	.0 (.0, 1.0)	6.0 (4.0, 7.0)	9.0 (6.0, 11.0)	<.001
Right‐sided CT score	6.0 (2.0, 9.0)	1.0 (.0, 1.5)	6.0 (4.0, 7.0)	9.0 (5.0, 10.0)	<.001
VAS score					
Nasal congestion	7.0 (5.0, 8.0)	3.0 (1.5, 5.0)	6.0 (6.0, 7.8)	8.0 (7.0, 9.0)	<.001
Runny nose	6.0 (2.0, 8.0)	1.0 (.0, 1.0)	7.5 (6.2, 8.0)	7.0 (6.0, 8.0)	<.001
Reduced smell	3.0 (.0, 5.0)	.0 (.0, .0)	4.0 (.8, 5.0)	4.5 (.0, 7.2)	<.001
Headache	.0 (.0, 3.0)	.0 (.0, .0)	2.5 (.0, 4.0)	2.0 (.0, 3.0)	.060
Peripheral blood EOS count	.1 (.0, .2)	.1 (.1, .2)	.1 (.0, .1)	.1 (.0, .2)	.359
Peripheral blood EOS percentage	.0 (.0, .0)	.0 (.0, .0)	.0 (.0, .0)	.0 (.0, .0)	.144
Total serum IGE	70.0 (17.7, 219.8)	82.8 (15.6, 223.0)	40.3 (13.3, 119.0)	114.0 (24.0, 228.0)	.661
Allergen assessment					.816
1	26 (63.4)	4 (66.7)	6 (54.5)	16 (66.7)	
2	10 (24.4)	1 (16.7)	3 (27.3)	6 (25.0)	
3	4 (9.8)	1 (16.7)	2 (18.2)	1 (4.2)	
4	1 (2.4)	0 (.0)	0 (.0)	1 (4.2)	
Atopic IGE					.687
Positive	19 (65.5)	2 (50.0)	6 (75.0)	11 (64.7)	
Negative	10 (34.5)	2 (50.0)	2 (25.0)	6 (35.3)	

Abbreviations: CRS, chronic rhinosinusitis; CRSwNP, CRS with nasal polyps; HC, healthy control.

**TABLE 2:** Regression analysis of ZFP36L1 and CAMK2A expression levels in chronic rhinosinusitis.

	*N*	Mean (SD)	*β* (95%CI)	*p*
ZFP36L1				
HC	16	5.6 (3.4)	0	
CRS	14	13.7 (3.2)	8.06 (4.29, 11.83)	<.001
CRSwNP	32	20.4 (6.5)	14.85 (11.69, 18.00)	<.001
*p* for trend				<.001
CAMK2A				
HC	16	20.5 (6.6)	0	
CRS	14	15.2 (4.7)	−5.30 (−9.15, −1.45)	<.01
CRSwNP	32	2.8 (3.2)	−17.77 (−21.09, −14.45)	<.001
*p* for trend				<.001

Abbreviations: CRS, chronic rhinosinusitis; CRSwNP, CRS with nasal polyps; HC, healthy control.

## 4 DISCUSSION

Accumulating evidence demonstrates that both RBPs and ferroptosis are among the important factors involved in CRS progression.^10^, ^11^ Furthermore, altered mitochondrial functions in epithelial cells have been implicated in CRSwNP pathogenesis.^15^ However, the precise regulatory mechanisms governing the interaction between RBPs and ferroptosis in CRS, as well as the role of RBP‐mediated mitochondrial regulation in disease pathogenesis, remain incompletely understood. In this study, we employed an integrated approach combining differential expression analysis, WGCNA and machine learning algorithms to systematically identify key genes associated with CRS development. We identified 6 hub genes from 30 candidate target genes: *BEX1, HMGCS2, HMOX1, LDHD, SLC25A48* and *ZFP36L1*, which are associated with critical cellular processes including apoptosis, epithelial–mesenchymal transition (EMT), immune‐related functions and the wnt/β‐catenin signalling pathway, central to CRS pathogenesis. Among these, *HMGCS2* and *HMOX1* have been previously reported to correlate with CRS pathology, serving as biomarkers for CRS and correlating with mitochondrial metabolism, immune cell infiltration and oxidative stress responses.^22^, ^23^, ^24^, ^25^ Most hub genes identified in our analysis have been shown to correlate with wnt/β‐catenin activity,^26^, ^27^, ^28^, ^29^, ^30^ suggesting that molecular biomarkers involved in CRS pathogenesis are significantly associated with wnt/β‐catenin pathway activity.

The mammalian ZFP36 family, also known as tristetraprolin, comprises CCCH‐type zinc‐finger proteins containing zinc‐finger domains that bind RNA and promote RNA degradation.^31^ Recent studies have established that ZFP36 family proteins regulate ferroptosis through multiple mechanisms in inflammatory diseases.^32^, ^33^ ZFP36 proteins modulate cellular sensitivity to ferroptosis by regulating ferroptosis‐related signalling pathways; for example, downregulation of ZFP36 triggers ferritin autophagy activation and ferroptosis initiation,^34^ whereas overexpression suppresses ferroptosis‐related markers.^35^ Zhang et al. characterized ZFP36 as a ferroptosis suppressor gene,^36^ and ZFP36 downregulation has been shown to enhance ferroptosis sensitivity in hepatic stellate cells.^37^ Moreover, Stockwell et al. demonstrated that ZFP36 modulates cellular antioxidant capacity by regulating GSH peroxidase activity.^38^ As CRS is an inflammatory disease closely associated with immune responses and ferroptosis, ZFP36L1 may regulate both inflammatory responses and ferroptosis by modulating immune cell function and activity. Cook et al. identified a critical role for ZFP36 proteins in regulating T cell quiescence,^39^ and Zhang et al. demonstrated that ZFP36 inactivation in mice results in a complex inflammatory syndrome characterized by increased TNF‐α production.^40^ Although the detailed mechanisms by which ZFP36L1 directly regulates mitochondrial function in CRS have not been fully characterized, Li et al. identified ZFP36 as a key mitochondrial dysfunction‐related prognostic biomarker,^41^ and ZFP36L1 may affect mitochondrial morphology, distribution and function by regulating genes or proteins associated with mitochondrial dynamics.^42^ In our study, OCR was significantly reduced and ECAR was significantly elevated in WT‐LPS groups, indicating inhibition of mitochondrial respiratory function. Notably, OCR in the ZFP36L1^KO‐LPS group showed improvement, whereas ECAR decreased compared to WT‐LPS, indicating that ZFP36L1 knockout partially alleviates CRS‐induced inhibition of mitochondrial respiration, consistent with its critical role in CRS pathogenesis.

To elucidate the downstream mechanisms of *ZFP36L1* in CRS, we identified calcium/calmodulin‐dependent protein kinase II alpha (*CAMK2A*) as a direct target of *ZFP36L1*‐mediated mRNA degradation. *CAMK2A* is a multifunctional serine/threonine kinase that plays pivotal roles in calcium signalling, synaptic plasticity and cellular metabolism. Importantly, accumulating evidence demonstrates that CAMK2A localizes to mitochondria and critically regulates mitochondrial function and quality control. Studies have shown that mitochondrial CaMKII controls mitochondrial metabolism, ATP production and ROS generation.^43^ Furthermore, Wu et al. demonstrated that CaMK signalling regulates mitochondrial biogenesis through PGC‐1α activation.^44^ Dysregulation of *CAMK2A* has been implicated in various diseases characterized by mitochondrial dysfunction and oxidative stress. Although *CAMK2A* has been extensively studied in neurons, its 3′‐UTR contains conserved AREs that are recognized by ZFP36 family proteins across different cell types. Our dual‐luciferase assays confirm that this regulatory mechanism operates in epithelial cells. *CAMK2A* may influence iron homeostasis through its regulation of calcium‐dependent signalling pathways that modulate ferritin expression and iron uptake. Disrupted calcium homeostasis due to *CAMK2A* deficiency could impair cellular iron buffering capacity, thereby increasing ferroptosis susceptibility.

Our comprehensive mechanistic studies established *CAMK2A* as a bona fide target of *ZFP36L1*. RIP assays demonstrated direct binding between ZFP36L1 protein and *CAMK2A* mRNA, whereas mRNA stability experiments showed that *ZFP36L1* promotes *CAMK2A* mRNA decay. Dual‐luciferase reporter assays further confirmed that *ZFP36L1* regulates *CAMK2A* through ARE in its 3′‐UTR, as mutation of these AREs abolished *ZFP36L1*‐mediated regulation. This post‐transcriptional regulatory mechanism is consistent with the well‐established function of ZFP36 family proteins in binding AREs and promoting target mRNA degradation. The negative correlation between *ZFP36L1* and *CAMK2A* expression observed in both animal models and clinical samples provides strong evidence for this regulatory axis. During chronic inflammation in CRS, elevated *ZFP36L1* expression leads to *CAMK2A* suppression, thereby impairing mitochondrial quality control systems in nasal epithelial cells. This represents a novel mechanism linking inflammatory responses to mitochondrial dysfunction and ferroptosis in CRS. The functional rescue experiments provided compelling evidence that *CAMK2A* acts as a critical downstream effector of ZFP36L1 in regulating ferroptosis and mitochondrial dysfunction. Knockdown of *CAMK2A* completely reversed the protective effects of *ZFP36L1* depletion on ferroptosis markers, ROS accumulation and mitochondrial morphology. This genetic epistasis experiment demonstrates that *CAMK2A* functions downstream of *ZFP36L1* in the regulatory hierarchy. Mechanistically, *CAMK2A* deficiency likely compromises mitochondrial calcium buffering capacity and impairs mitochondrial quality control mechanisms. Studies have demonstrated that mitochondria play a central role in ferroptosis through multiple mechanisms, including ROS generation, lipid peroxidation and energy metabolism.^45^ Mitochondrial dysfunction leads to excessive ROS production, which promotes lipid peroxidation and ferroptosis.^46^ Furthermore, disruption of mitochondrial calcium homeostasis, which is regulated by CAMK2A, can exacerbate oxidative stress and ferroptosis susceptibility.^47^ The complete rescue of the ZFP36L1 knockdown phenotype by CAMK2A depletion establishes the ZFP36L1–CAMK2A axis as a key regulatory pathway in CRS‐associated ferroptosis and mitochondrial dysfunction.

Our findings demonstrate that mitochondrial respiration is involved in ferroptosis in CRS: intervention with ferroptosis inhibitors or inducers significantly altered mitochondrial functions, including structure, quantity, membrane potential, cytochrome *c* oxidase activity and ATP levels. During ferroptosis, ferroptosis can lead to changes in mitochondrial morphology,^48^ and our research indicates that mitochondria become smaller with increased membrane density, decreased cristae and outer membrane ruptures. Ferroptosis is closely related to mitochondrial energy metabolism; during ferroptosis, mitochondrial energy metabolism is disrupted, leading to decreased ATP production.^49^ Furthermore, ferroptosis is related to the imbalance between mitochondrial ROS production and antioxidant defense.^38^, ^50^

From a translational perspective, the *ZFP36L1–CAMK2A*‐ferroptosis axis represents a promising therapeutic target for CRS. Our clinical validation demonstrated that *ZFP36L1* expression progressively increases with disease severity, inversely correlating with *CAMK2A* levels and positively associating with ferroptosis markers (ROS, Fe^2+^). These findings suggest that therapeutic strategies aimed at modulating this axis could provide clinical benefits. Ferroptosis has emerged as an important therapeutic target in various inflammatory diseases, and pharmacological modulation of ferroptosis pathways has shown promise in preclinical studies.^38^ Potential approaches include (1) pharmacological inhibition of *ZFP36L1* to restore *CAMK2A* expression; (2) direct pharmacological activation of *CAMK2A* or enhancement of calcium signalling pathways; (3) administration of ferroptosis inhibitors such as ferrostatin‐1 or liproxstatin‐1 to mitigate downstream cellular damage^51^; or (4) enhancement of mitochondrial quality control mechanisms through PGC‐1α activators or mitochondrial‐targeted antioxidants. Small molecule inhibitors targeting RBP‐mRNA interactions have been successfully developed for other systems and could be adapted for the *ZFP36L1–CAMK2A* axis. Given the central role of oxidative stress in CRS pathogenesis, combination therapies targeting both ferroptosis and inflammation may provide synergistic benefits. Future studies should investigate whether these strategies can ameliorate CRS symptoms in preclinical models and evaluate their safety and efficacy in clinical trials.

Despite these significant findings, our study has several limitations. First, although we identified the *ZFP36L1–CAMK2A* axis as central to CRS pathogenesis, the complete signalling network and potential compensatory mechanisms remain to be fully characterized. Second, although our rescue experiments strongly support *CAMK2A* as the key mediator, *ZFP36L1* likely regulates multiple target mRNAs simultaneously, and the relative contributions of these targets warrant further investigation. Third, our clinical cohort, while providing valuable validation, was relatively limited in size. Larger clinical cohorts are needed to confirm the generalizability of our findings. Fourth, we focused primarily on epithelial cells, but CRS involves complex interactions among multiple cell types, including immune cells, fibroblasts and endothelial cells. The role of the *ZFP36L1–CAMK2A* axis in these other cell types requires further exploration. Fifth, our in vitro mechanistic studies were conducted using immortalized nasal epithelial cell lines. Although these cell lines are widely used and provide reproducible results, they may not fully recapitulate the physiological characteristics of primary human nasal epithelial cells. Sixth, our animal experiments were conducted exclusively in male mice, which may limit the translational applicability of our findings to both sexes. This design choice was based on previous CRS modelling studies that predominantly used male mice due to potential hormonal influences on inflammatory responses. However, we acknowledge that this approach does not meet current NIH guidelines recommending balanced sex representation. Future investigations should include female mice to determine whether sex‐specific differences exist in the ZFP36L1–CAMK2A‐ferroptosis axis in CRS. Finally, although we demonstrated therapeutic potential, actual therapeutic interventions targeting this axis need to be developed and tested in preclinical and clinical settings.

## 5 CONCLUSION

Among multiple regulatory mechanisms, this study reveals a novel *ZFP36L1–CAMK2A* axis as one of the regulatory mechanisms in CRS pathogenesis. *ZFP36L1* directly binds and destabilizes *CAMK2A* mRNA through ARE‐mediated mechanisms, leading to mitochondrial dysfunction and enhanced ferroptosis in nasal epithelial cells. Functional rescue experiments establish *CAMK2A* as a critical downstream effector of *ZFP36L1* in this pathway. Clinical validation confirms that *ZFP36L1* expression inversely correlates with *CAMK2A* levels, and both associate with disease severity. These findings provide new mechanistic insights into CRS progression and identify the *ZFP36L1–CAMK2A* axis as a potential therapeutic target for future drug development.

## AUTHOR CONTRIBUTIONS

Jiayi Xiong and Shihan Zhang conceived and designed the study, carried out the experiments, analysed the data and drafted the original manuscript. Hongbing Liu contributed to the methodology, supervised data collection and critically reviewed and edited the intellectual content. Chunhua Li and Yufeng Ai assisted with the experimental setup, performed data collection and participated in the interpretation of results. Xinru Liu and Yuxiang Liu were responsible for data analysis and the creation of figures and tables. All authors contributed to the writing process; specifically, Hongbing Liu led the introduction and methods sections, whereas the results and discussion were the focus of the other authors. All authors have reviewed and approved the final manuscript.

## CONFLICT OF INTEREST STATEMENT

The authors declare no conflicts of interest.

## ETHICS STATEMENT

This study was approved by the Second Affiliated Hospital of Nanchang University Jiangxi Medical College ethical review committee (NCULAE‐20221031181, O‐Ethical Review of Medical Research [2023] No. [03]). All of the patients signed the informed consent form.

## Supporting information



Supporting Information

Supporting Information

Supporting Information

Supporting Information

Supporting Information

Supporting Information

## Data Availability

Data sharing is not applicable to this article as no datasets were generated or analysed during the current study.

## References

[1] Tsuda T , Suzuki M , Kato Y , et al. The current findings in eosinophilic chronic rhinosinusitis. Auris Nasus Larynx. 2024;51(1):51‐60. 37574421 10.1016/j.anl.2023.08.002

[2] Cao PP , Wang ZC , Schleimer RP , et al. Pathophysiologic mechanisms of chronic rhinosinusitis and their roles in emerging disease endotypes. Ann Allergy Asthma Immunol. 2019;122(1):33‐40. 30326322 10.1016/j.anai.2018.10.014 PMC6309633

[3] Gebauer F , Schwarzl T , Valcárcel J , et al. RNA‐binding proteins in human genetic disease. Nat Rev Genet. 2021;22(3):185‐198. 33235359 10.1038/s41576-020-00302-y

[4] Shi LL , Ma J , Deng YK , et al. Cold‐inducible RNA‐binding protein contributes to tissue remodeling in chronic rhinosinusitis with nasal polyps. Allergy. 2021;76(2):497‐509. 32198936 10.1111/all.14287

[5] Zhipu N , Zitao H , Jichao S , et al. Research advances in roles of microRNAs in nasal polyp. Front Genet. 2022;13:1043888. 36506304 10.3389/fgene.2022.1043888 PMC9732428

[6] Li HX , Fei J , Xu W , et al. The characterization and validation of regulated cell death‐related genes in chronic rhinosinusitis with nasal polyps. Int Immunopharmacol. 2025;154:114509. 40158428 10.1016/j.intimp.2025.114509

[7] Huang GJ , Liu HB . Identification and validation of ferroptosis‐related genes for chronic rhinosinusitis with nasal polyps. Eur Arch Otorhinolaryngol. 2023;280(3):1501‐1508. 36255469 10.1007/s00405-022-07696-x

[8] Li P , Jiang M , Li K , et al. Glutathione peroxidase 4‐regulated neutrophil ferroptosis induces systemic autoimmunity. Nat Immunol. 2021;22(9):1107‐1117. 34385713 10.1038/s41590-021-00993-3 PMC8609402

[9] Cao L , Wang Y , Liu J , et al. Long non‐coding RNA TPT1‐AS1 inhibits ferroptosis in ovarian cancer by regulating GPX4 via CREB1 regulation. Am J Reprod Immunol. 2024;92(2):e13864. 39141012 10.1111/aji.13864

[10] Wang Q , Guo Y , Wang W , et al. RNA binding protein DAZAP1 promotes HCC progression and regulates ferroptosis by interacting with SLC7A11 mRNA. Exp Cell Res. 2021;399(1):112453. 33358859 10.1016/j.yexcr.2020.112453

[11] Chen X , Yang C , Wang W , et al. Exploration of prognostic genes and risk signature in breast cancer patients based on RNA binding proteins associated with ferroptosis. Front Genet. 2023;14:1025163. 36911389 10.3389/fgene.2023.1025163 PMC9998954

[12] Ma J , Sun L , Gao W , et al. RNA binding protein: coordinated expression between the nuclear and mitochondrial genomes in tumors. J Transl Med. 2023;21(1):512. 37507746 10.1186/s12967-023-04373-3 PMC10386658

[13] Klein K , He K , Younes AI , et al. Role of mitochondria in cancer immune evasion and potential therapeutic approaches. Front Immunol. 2020;11:573326. 33178201 10.3389/fimmu.2020.573326 PMC7596324

[14] Mortazavi Farsani SS , Soni J , Jin L , et al. Pyruvate kinase M2 activation reprograms mitochondria in CD8 T cells, enhancing effector functions and efficacy of anti‐PD1 therapy. Cell Metab. 2025;37(6):1294‐1310. 40199327 10.1016/j.cmet.2025.03.003 PMC12137016

[15] Yoon YH , Yeon SH , Choi MR , et al. Altered mitochondrial functions and morphologies in epithelial cells are associated with pathogenesis of chronic rhinosinusitis with nasal polyps. Allergy Asthma Immunol Res. 2020;12(4): 653‐668. 32400131 10.4168/aair.2020.12.4.653 PMC7224996

[16] Wang C , Zhou ML , Liu YC , et al. The roles of autophagy, mitophagy, and the Akt/mTOR pathway in the pathogenesis of chronic rhinosinusitis with nasal polyps. J Immunol Res. 2022;2022:2273121. 35747690 10.1155/2022/2273121 PMC9213180

[17] Zhao Y , Li M , Yang Y , et al. Identification of macrophage polarization‐related genes as biomarkers of chronic obstructive pulmonary disease based on bioinformatics analyses. Biomed Res Int. 2021;2021:9921012. 34250093 10.1155/2021/9921012 PMC8238569

[18] Ritchie ME , Phipson B , Wu D , et al. Limma powers differential expression analyses for RNA‐sequencing and microarray studies. Nucleic Acids Res. 2015;43(7):e47. 25605792 10.1093/nar/gkv007 PMC4402510

[19] Langfelder P , Horvath S . WGCNA: an R package for weighted correlation network analysis. BMC Bioinformatics. 2008;9:559. 19114008 10.1186/1471-2105-9-559 PMC2631488

[20] Friedmann Angeli JP , Schneider M , Proneth B , et al. Inactivation of the ferroptosis regulator Gpx4 triggers acute renal failure in mice. Nat Cell Biol. 2014;16(12):1180‐1191. 25402683 10.1038/ncb3064 PMC4894846

[21] Rigden DJ , Fernández XM . The 2021 nucleic acids research database issue and the online molecular biology database collection. Nucleic Acids Res. 2021;49(D1):D1‐D9. 33396976 10.1093/nar/gkaa1216 PMC7778882

[22] Yang B , Gu M , Hong C , et al. Integrated machine learning and bioinformatic analysis of mitochondrial‐related signature in chronic rhinosinusitis with nasal polyps. World Allergy Organ J. 2024;17(10):100964. 39328210 10.1016/j.waojou.2024.100964 PMC11426132

[23] Tai J , Shin JM , Park J , et al. Oxidative stress and antioxidants in chronic rhinosinusitis with nasal polyps. Antioxidants (Basel). 2023;12(1):195. 36671057 10.3390/antiox12010195 PMC9854928

[24] Wang E , Li S , Li Y , et al. HMOX1 as a potential drug target for upper and lower airway diseases: insights from multi‐omics analysis. Respir Res. 2025;26(1):41. 39871287 10.1186/s12931-025-03124-w PMC11773792

[25] Zi J , Yu L , Wang L , et al. Identification and validation of autophagy‐related genes and exploration of their relationship with disease severity in chronic rhinosinusitis with nasal polyps. Asia Pac Allergy. 2024;14(4):162‐173. 39624449 10.5415/apallergy.0000000000000159 PMC11608634

[26] Wang Q , Liang N , Yang T , et al. DNMT1‐mediated methylation of BEX1 regulates stemness and tumorigenicity in liver cancer. J Hepatol. 2021;75(5):1142‐1153. 34217777 10.1016/j.jhep.2021.06.025

[27] Kim JT , Li C , Weiss HL , et al. Regulation of ketogenic enzyme HMGCS2 by Wnt/β‐catenin/PPARγ pathway in intestinal cells. Cells. 2019;8(9):1106. 31546785 10.3390/cells8091106 PMC6770209

[28] Xue B , Yan L , Ye M , et al. PROTAC‐surufatinib suppresses pancreatic neuroendocrine neoplasms progression by inducing ferroptosis through inhibiting WNT/β‐catenin pathway mediated by HMOX1. Int J Biol Sci. 2025;21(6):2476‐2492. 40303295 10.7150/ijbs.106357 PMC12035883

[29] Wang H , Zhang Y , Du S . Integrated analysis of lactate‐related genes identifies POLRMT as a novel marker promoting the proliferation, migration and energy metabolism of hepatocellular carcinoma via Wnt/β‐catenin signaling. Am J Cancer Res. 2024;14(3):1316‐1337. 38590398 10.62347/ZTTG4319 PMC10998737

[30] Wei X , Deng W , Dong Z , et al. Identification of subtypes and a delayed graft function predictive signature based on ferroptosis in renal ischemia‐reperfusion injury. Front Cell Dev Biol. 2022;10:800650. 35211472 10.3389/fcell.2022.800650 PMC8861527

[31] Rynne J , Ortiz‐Zapater E , Bagley D C , et al. The RNA binding proteins ZFP36L1 and ZFP36L2 are dysregulated in airway epithelium in human and a murine model of asthma. Front Cell Dev Biol. 2023;11:1241008. 37928904 10.3389/fcell.2023.1241008 PMC10624177

[32] Fan J , Zhu T , Tian X , et al. Exploration of ferroptosis and necroptosis‐related genes and potential molecular mechanisms in psoriasis and atherosclerosis. Front Immunol. 2024;15:1372303. 39072329 10.3389/fimmu.2024.1372303 PMC11272566

[33] Wufuer D , Li Y , Aierken H , et al. Bioinformatics‐led discovery of ferroptosis‐associated diagnostic biomarkers and molecule subtypes for tuberculosis patients. Eur J Med Res. 2023;28(1):445. 37853432 10.1186/s40001-023-01371-5 PMC10585777

[34] Jiao Y , Liu X , Shi J , et al. Unraveling the interplay of ferroptosis and immune dysregulation in diabetic kidney disease: a comprehensive molecular analysis. Diabetol Metab Syndr. 2024;16(1):86. 38643193 10.1186/s13098-024-01316-w PMC11032000

[35] Li X , Hu Y , Wu Y , et al. Exosomal let‐7a‐5p derived from human umbilical cord mesenchymal stem cells alleviates coxsackievirus B3‐induced cardiomyocyte ferroptosis via the SMAD2/ZFP36 signal axis. J Zhejiang Univ Sci B. 2024;25(5):422‐437. 38725341 10.1631/jzus.B2300077 PMC11087186

[36] Ma J , Li C , Liu T , et al. Identification of markers for diagnosis and treatment of diabetic kidney disease based on the ferroptosis and immune. Oxid Med Cell Longev. 2022;2022:9957172. 36466094 10.1155/2022/9957172 PMC9712001

[37] Zhang Z , Guo M , Li Y , et al. RNA‐binding protein ZFP36/TTP protects against ferroptosis by regulating autophagy signaling pathway in hepatic stellate cells. Autophagy. 2020;16(8):1482‐1505. 31679460 10.1080/15548627.2019.1687985 PMC7469536

[38] Stockwell BR . Ferroptosis turns 10: emerging mechanisms, physiological functions, and therapeutic applications. Cell. 2022;185(14):2401‐2421. 35803244 10.1016/j.cell.2022.06.003 PMC9273022

[39] Cook ME , Bradstreet TR , Webber AM , et al. The ZFP36 family of RNA binding proteins regulates homeostatic and autoreactive T cell responses. Sci Immunol. 2022;7(76):eabo0981. 36269839 10.1126/sciimmunol.abo0981 PMC9832469

[40] Zhang Y , Li NF , Abulikemu S , et al. Relationship between zinc finger protein 36 (ZFP36) gene polymorphisms and obstructive sleep apnea. Genet Mol Res. 2015;14(2):6733‐6743. 26125882 10.4238/2015.June.18.17

[41] Li Y , CUI Y , Wang Z , et al. Development and validation of a hypoxia‐ and mitochondrial dysfunction‐ related prognostic model based on integrated single‐cell and bulk RNA sequencing analyses in gastric cancer. Front Immunol. 2024;15:1419133. 39165353 10.3389/fimmu.2024.1419133 PMC11333257

[42] Guo H , Jiang Y , Gu Z , et al. ZFP36 protects against oxygen‐glucose deprivation/reoxygenation‐induced mitochondrial fragmentation and neuronal apoptosis through inhibiting NOX4‐DRP1 pathway. Brain Res Bull. 2022;179:57‐67. 34896479 10.1016/j.brainresbull.2021.12.003

[43] Luczak ED , Wu Y , Granger JM , et al. Mitochondrial CaMKII causes adverse metabolic reprogramming and dilated cardiomyopathy. Nat Commun. 2020;11(1):4416. 32887881 10.1038/s41467-020-18165-6 PMC7473864

[44] Wu H , Kanatous SB , Thurmond FA , et al. Regulation of mitochondrial biogenesis in skeletal muscle by CaMK. Science. 2002;296(5566):349‐352. 11951046 10.1126/science.1071163

[45] Gao M , Yi J , Zhu J , et al. Role of mitochondria in ferroptosis. Mol Cell. 2019;73(2):354‐363. 30581146 10.1016/j.molcel.2018.10.042 PMC6338496

[46] Kuang F , Liu J , Tang D , et al. Oxidative damage and antioxidant defense in ferroptosis. Front Cell Dev Biol. 2020;8:586578. 33043019 10.3389/fcell.2020.586578 PMC7527737

[47] Bertero E , Maack C . Calcium signaling and reactive oxygen species in mitochondria. Circ Res. 2018;122(10):1460‐1478. 29748369 10.1161/CIRCRESAHA.118.310082

[48] Zhao K , He B , Xue K , et al. IL6ST: a novel therapeutic target for managing and treating colorectal cancer via ferroptosis. Turk J Gastroenterol. 2024;35(9):690‐698. 39344518 10.5152/tjg.2024.23353 PMC11391227

[49] Sun Q , Tu K , Xu Q , et al. Berberine suppresses colorectal cancer progression by inducing ferroptosis‐mediated energy metabolism disorders. J Adv Res. 2025;S2090‐1232(25):820–823. 10.1016/j.jare.2025.10.025 PMC13316491 41139018

[50] Niu B , Liao K , Zhou Y , et al. Application of glutathione depletion in cancer therapy: enhanced ROS‐based therapy, ferroptosis, and chemotherapy. Biomaterials. 2021;277:121110. 34482088 10.1016/j.biomaterials.2021.121110

[51] Skouta R , Dixon SJ , Wang J , et al. Ferrostatins inhibit oxidative lipid damage and cell death in diverse disease models. J Am Chem Soc. 2014;136(12):4551‐4556. 24592866 10.1021/ja411006a PMC3985476

