# Supplementary figures and images for: RNA binding protein ZFP36L1 promotes ferroptosis in chronic rhinosinusitis by destabilizing CAMK2A mRNA and impairing mitochondrial quality control
Source: Clin Transl Med. 2026 Apr 29;16(5):e70661. doi: 10.1002/ctm2.70661 (PMC13125952; doi:10.1002/ctm2.70661)

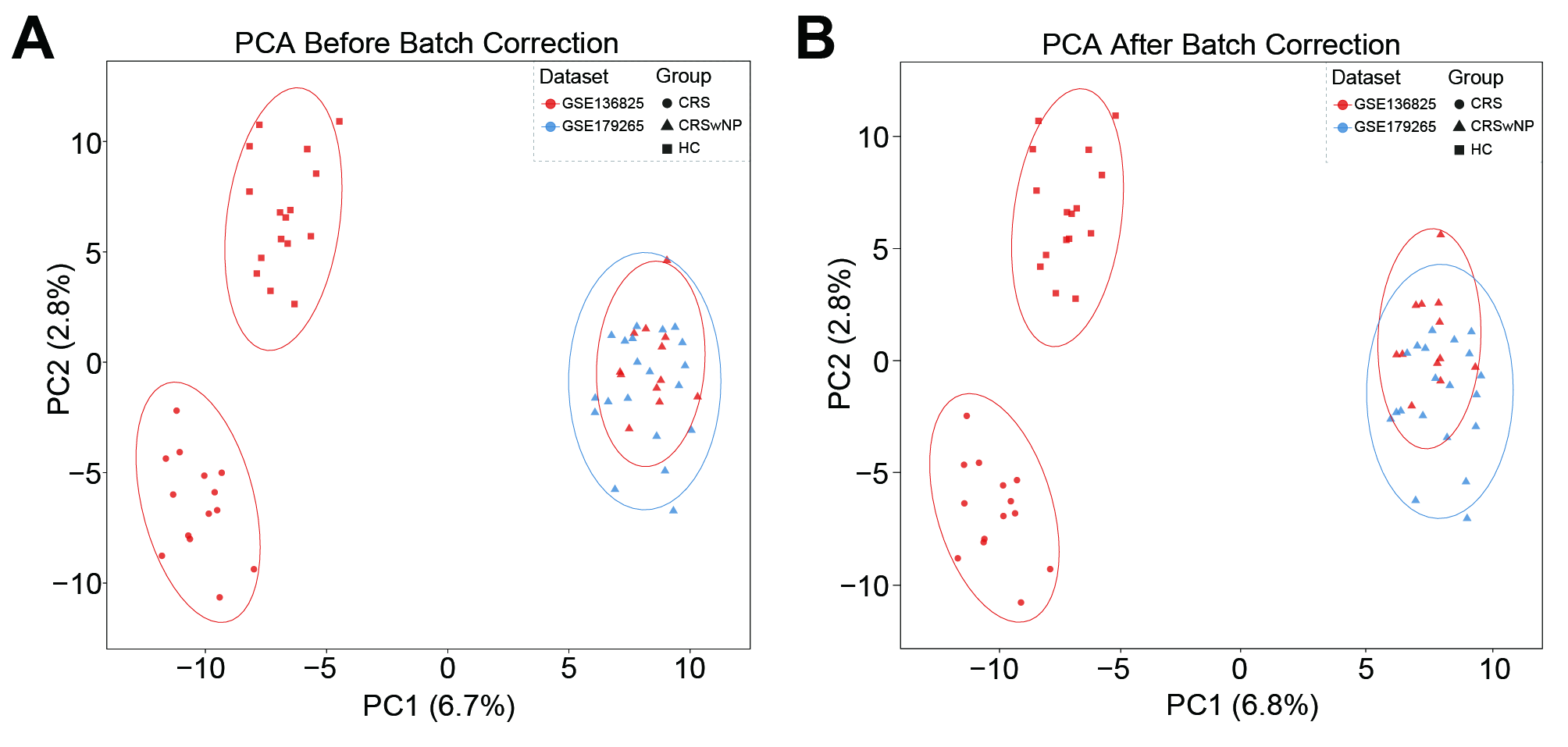

Supplement: Supplementary file 1 — Supporting Information [file CTM2-16-e70661-s003.tif]

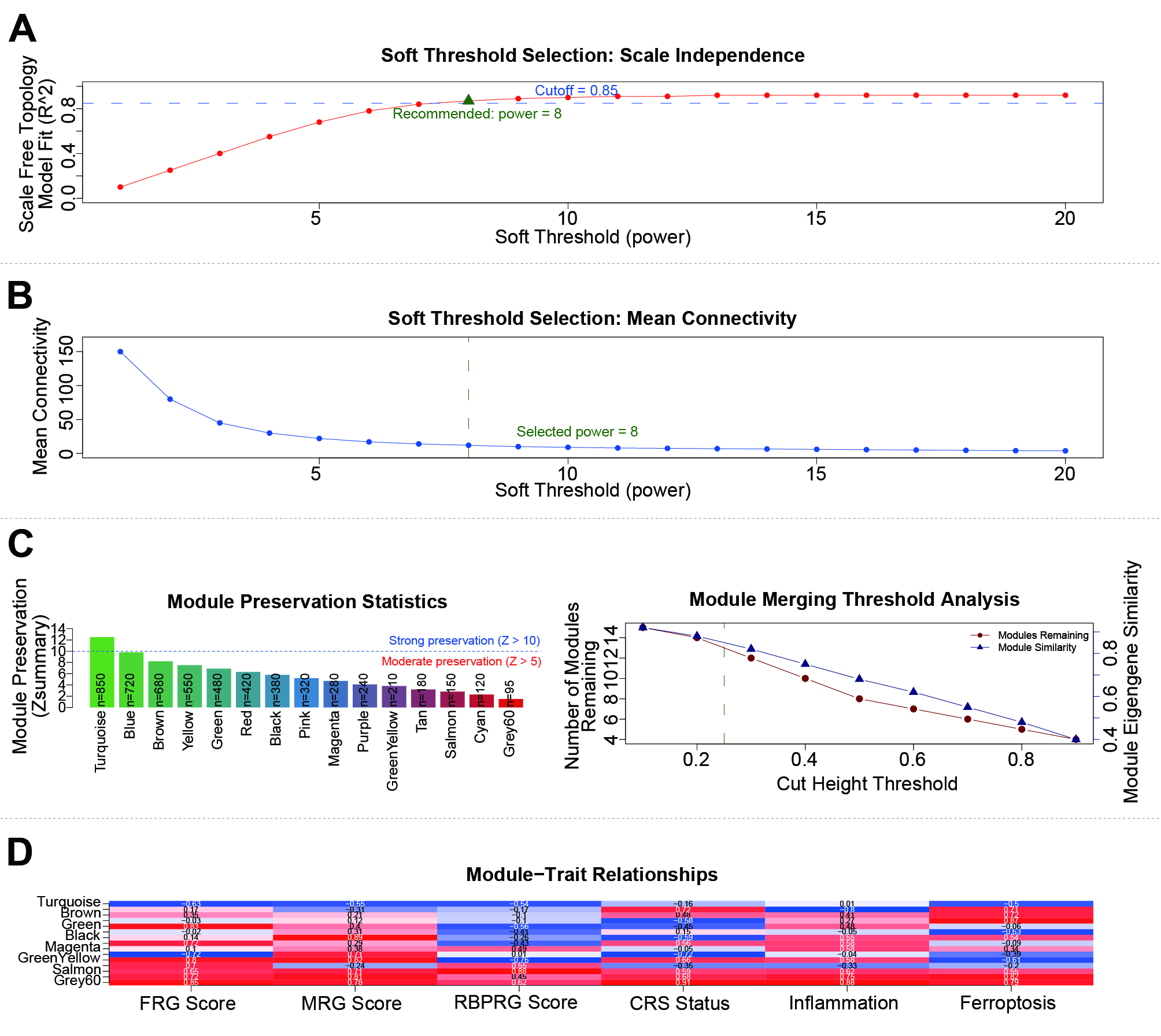

Supplement: Supplementary file 2 — Supporting Information [file CTM2-16-e70661-s005.tif]

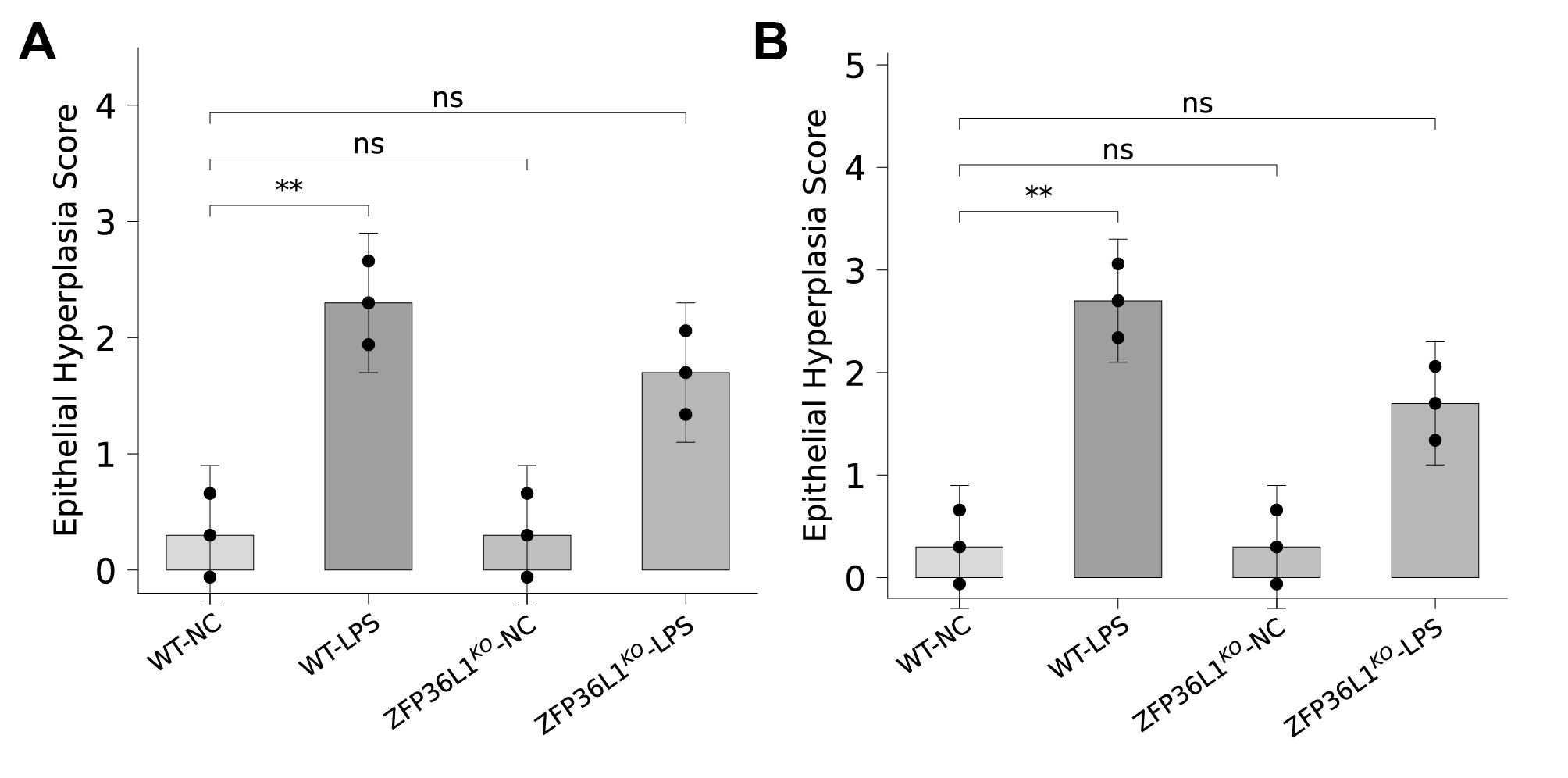

Supplement: Supplementary file 3 — Supporting Information [file CTM2-16-e70661-s004.tif]

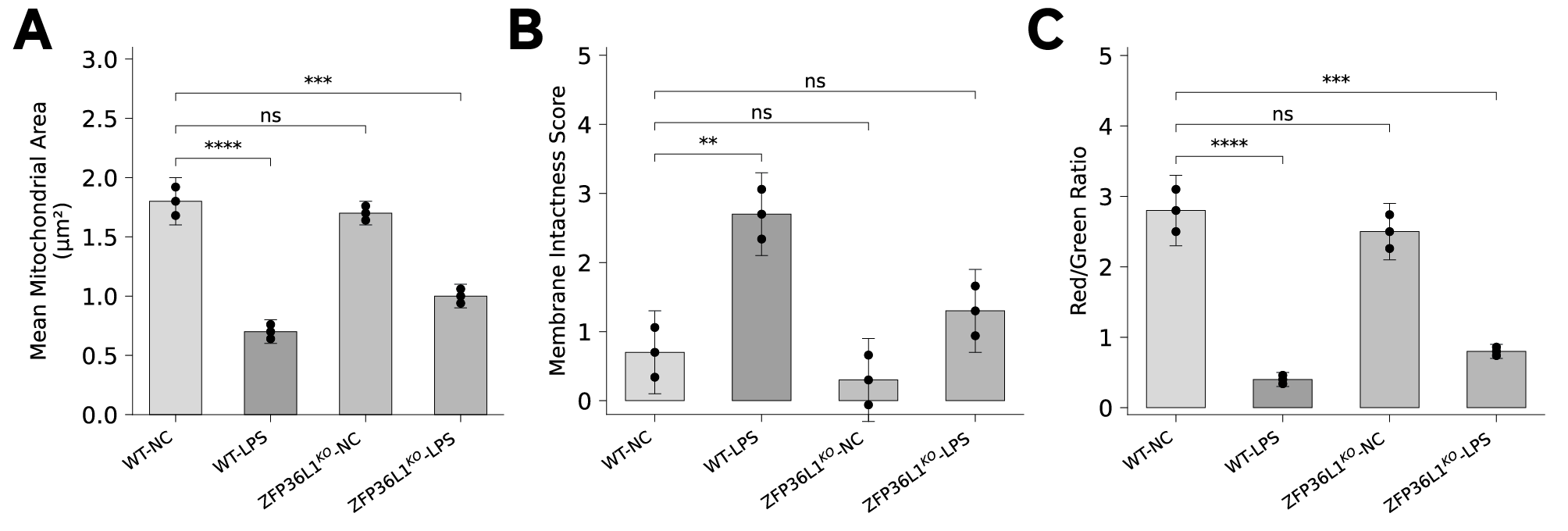

Supplement: Supplementary file 4 — Supporting Information [file CTM2-16-e70661-s002.tif]

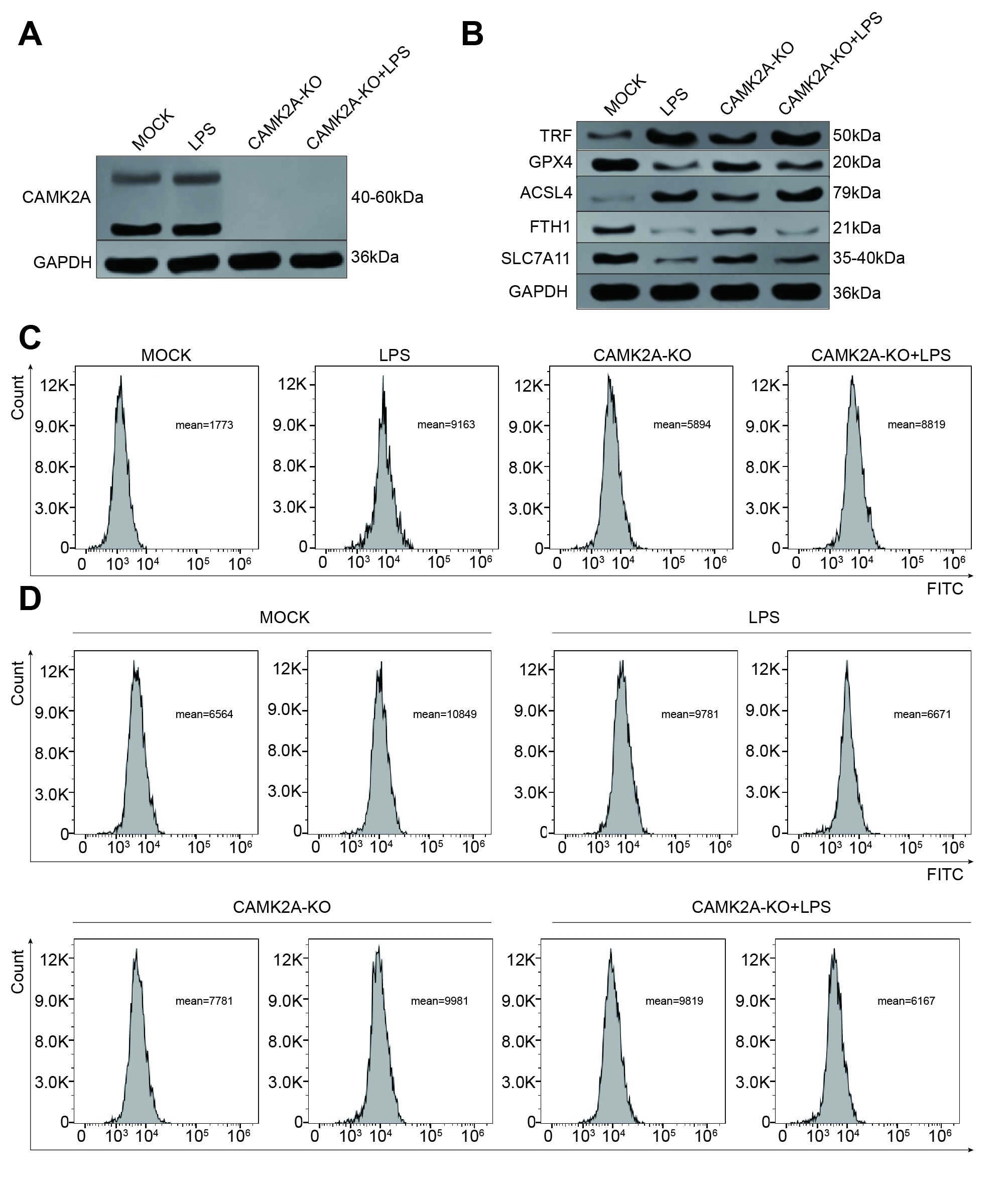

Supplement: Supplementary file 5 — Supporting Information [file CTM2-16-e70661-s001.tif]

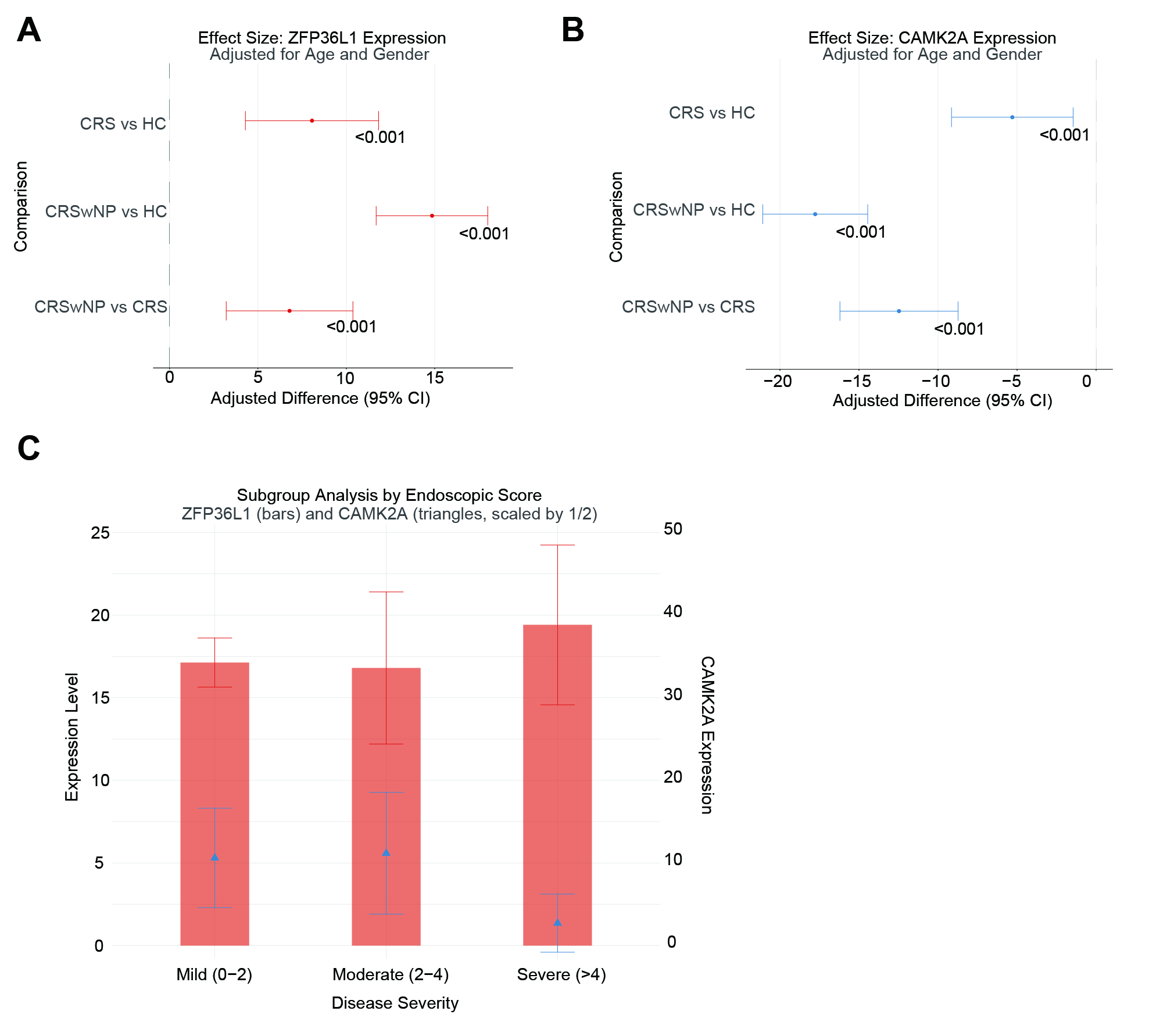

Supplement: Supplementary file 6 — Supporting Information [file CTM2-16-e70661-s006.tif]
